# Systemic Lupus Erythematosus Patients with DNASE1L3·Deficiency Have a Distinctive and Specific Genic Circular DNA Profile in Plasma

**DOI:** 10.3390/cells12071061

**Published:** 2023-03-31

**Authors:** Daniela Gerovska, Marcos J. Araúzo-Bravo

**Affiliations:** 1 Computational Biology and Systems Biomedicine, Biodonostia Health Research Institute, Calle Doctor Begiristain s/n, 20014 San Sebastian, Spain; 2 Basque Foundation for Science, IKERBASQUE, Calle María Díaz Harokoa 3, 48013 Bilbao, Spain; 3 Max Planck Institute for Molecular Biomedicine, Computational Biology and Bioinformatics, Roentgenstr. 20, 48149 Muenster, Germany; 4 Department of Cell Biology and Histology, Faculty of Medicine and Nursing, University of Basque Country (UPV/EHU), 48940 Leioa, Spain

**Keywords:** extrachromosomal, circular DNA, eccDNA, differential, DNASE1L3, systemic lupus erythematosus, autoimmune, rheumatic, glomerulonephritis

## Abstract

Cell-free (cf) extrachromosomal circular DNA (eccDNA) has a potential clinical application as a biomarker. Systemic lupus erythematosus (SLE) is a systemic autoimmune disease with a complex immunological pathogenesis, associated with autoantibody synthesis. A previous study found that SLE patients with deoxyribonuclease 1-like 3 (DNASE1L3) deficiency exhibit changes in the frequency of short and long eccDNA in plasma compared to controls. Here, using the DifCir method for differential analysis of short-read sequenced purified eccDNA data based on the split-read signal of the eccDNA on circulomics data, we show that SLE patients with DNASE1L3 deficiency have a distinctive profile of eccDNA excised by gene regions compared to controls. Moreover, this profile is specific; cf-eccDNA from the top 93 genes is detected in all SLE with DNASE1L3 deficiency samples, and none in the control plasma. The top protein coding gene producing eccDNA-carrying gene fragments is the transcription factor *BARX2,* which is involved in skeletal muscle morphogenesis and connective tissue development. The top gene ontology terms are ‘positive regulation of torc1 signaling’ and ‘chondrocyte development’. The top Harmonizome terms are ‘lymphopenia’, ‘metabolic syndrome x’, ‘asthma’, ‘cardiovascular system disease‘, ‘leukemia’, and ‘immune system disease’. Here, we show that gene associations of cf-eccDNA can serve as a biomarker in the autoimmune rheumatic diseases.

## 1. Introduction

Systemic lupus erythematosus (SLE) is a highly prevalent human autoimmune rheumatic disease characterized by abnormal T cells, overactive B cells, and the production of large amounts of various pathogenic autoantibodies. SLE causes progressive glomerulonephritis, arthritis, and an erythematous rash. Anti-DNA autoantibodies, and specifically anti-double stranded deoxyribonucleic acid (dsDNA) antibodies, are pathogenic in SLE, while cell-free chromatin-associated long DNA fragments are antigens for anti-DNA antibodies. Although it is still under discussion how and who triggers the production of DNA antibodies in SLE, there is growing evidence that failure in the clearance of cell-free DNA (cfDNA) by deoxyribonucleases (DNASES), and in particular by deoxyribonuclease 1-like 3 (DNASE1L3), can lead to the production of anti-DNA antibodies and SLE [1]. CfDNA is released into blood due to cell death, including necrosis, apoptosis, neutrophil extracellular net formation (NETosis), and pyroptosis, as well as by live cells; then, DNA fragmentation factor B (DFFB) cleaves dsDNA at internucleosome linker regions in chromatin into higher molecular weight DNA fragments, and then into oligonucleosomal fragments; and finally, cfDNA fragments are cleared by DNASES, DNASE1 (for <150 bp fragments), and DNASE1L3 (for larger and chromatin associated DNA) in healthy individuals, while in SLE, cfDNA is accumulated. The defective clearance of long fragments of cfDNA in SLE is largely attributed to impaired deoxyribonuclease 1-like 3 (DNASE1L3) [1].

Cell-free (cf) extrachromosomal circular DNA (cf-eccDNA) is a DNA molecule gaining increasing research interest as a biomarker with clinical applications, thanks to improvements in the eccDNA purification methods and the increased generation of circulomics data. Sin et al. [2] studied the effect of DNASE1L3 on the abundance and size distribution of the cf-eccDNA in SLE patients with DNASE1L3 mutations, and concluded that DNASE1L deficiency in humans leads to lengthening of the eccDNA in plasma. Multiple circulomics approaches have focused on discovering markers based on global features of the eccDNA, such as difference in their length and number between control and treatment conditions. Such global differences lack high sensitivity to distinguish between sample groups, and this problem is even more exacerbated in the case of using plasma samples. To improve the sensitivity of the circulomics analysis, we developed a computational method for differential analysis of eccDNA (DifCir) based on quantifying the number of produced per gene eccDNA (PpGCs) [3]. In a case when neither the transcriptomics methods nor the circulomics methods, based on measuring the abundance and frequency distribution of lengths of the eccDNA, were able to detect differences between sedentary and active skeletal muscle tissue of aged males, DifCir was able to detect very distinctive genic eccDNA profiles. 

**Figure 1:**
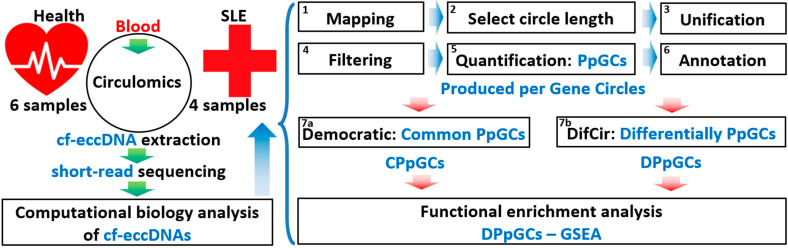
Experimental setup and computational analysis workflow of circulomics data of cf-eccDNA in systemic lupus erythematosus (SLE) and healthy control (HC) from Sin et al. [2]. Isolation and purification of circular DNA from plasma of SLE and HC [2] and subsequent assembly, annotation, quantification of eccDNA species, quantification of produced per gene circles (PpGCs), and calculation of differentially PpGCs (DPpGCs). Functional enrichment analysis of the DPpGCs performed with Harmonizome and GSEA.

Here, using the DifCir method on the circulomics data of Sin et al. [2], we show that SLE patients with DNASE1L3 loss-of-function mutation have a distinctive profile of cf-eccDNA excised by gene regions compared to healthy controls; thus, we propose that not only abundance and size distributions, but also specific genic cf-eccDNA profiles can be a biomarker in SLE activity. The experimental setup and the computational workflow we designed to find cf-eccDNA biomarkers for SLE with DNASE1L3 loss-of-function are shown in Figure 1.

## 2. Methods

### 2.1. Patient Characteristics

Four healthy human participants were recruited from the Department of Chemical Pathology, the Chinese University of Hong Kong (CUHK), with written informed consent. Three human participants with DNASE1L3 mutations were recruited from the Istituto Giannina Gaslini, also with written informed consent. Four blood samples in total were obtained from this patient cohort. Table 1 shows a summary of the patient characteristics based on the description provided in Sin et al. [2].

### 2.2. Sample Description

We used 6 control samples from the four healthy control (HC) participants, namely HC1, HC2-1, HC2-2, HC3-1, HC3-2, and HC4. The four data samples from the three SLE patients are denoted as SLE-1, SLE-2, SLE-3, and SLE-4, as provided by the CUHK Circulating Nucleic Acids Research Group and reported by Sin et al. [2].

### 2.3. Data Preprocessing

We used Trimmomatic-0.38 [4] to trim the adaptors from the 2 × 150 bp paired-end read sequenced data from Sin et al. [2].

### 2.4. EccDNA Mapping and Quantification of the Produced per Gene eccDNA (PpGCs)

We mapped the eccDNA using Circle_finder [5], using as arguments the hg38 built of the genome and minNonOverlap between two split reads equal to 10. The mapped reads were ranging from 55% to 100% of the total reads among the 10 samples (Table 2). We filtered out mitochondrial eccDNA and eccDNA with lengths greater than *L_max_* = 10 Kb. Next, we merged clusters of circles within a distance smaller than *D_min_* = 10 b and added up the split reads of the merged circles. We filtered out the circles with less than *JT_min_* = 2 split reads. We annotated the circles using bedtools intersect [6] on the resulting eccDNA bed files with a GencodeHuman38 bed file with the gene coordinates. Then, we unified the circles based on genes, i.e., the split reads of all the eccDNA that carry the same gene or fragment of this gene were added up to obtain unscaled produced per gene eccDNA (PpGC*_i_*) for each gene *i*. Next, we scaled by gene length, multiplying each PpGC*_i_* by a scale factor *L_Max_*/*L_i_*, where *L_Max_* is the length of the longest gene found in the dataset, and *L_i_* is the length of the gene *i*. Finally, we performed data equalization by the log_2_(PpGC + 1) transform of the quantified PpGCs to obtain the final PpGCs.

### 2.5. Differential Analysis for Identifying Differentially Produced per Gene DNA Circles (DPpGCs)

We calculated the average values for each group, HC and SLE. We selected the DPpGCs whose absolute value of difference of mean values between the two groups was less than a selection threshold *θ*_DPpGC_ = 5-fold change (FC) in log_2_ scale. We selected the statistically significant DPpGCs using the Student’s *t*-test, with a significance threshold α_DPpGC_ = 0.01.

## 3. Results

### 3.1. Abundance of Unique cf-eccDNA Is Higher in SLE Than in HC

**Figure 2:**
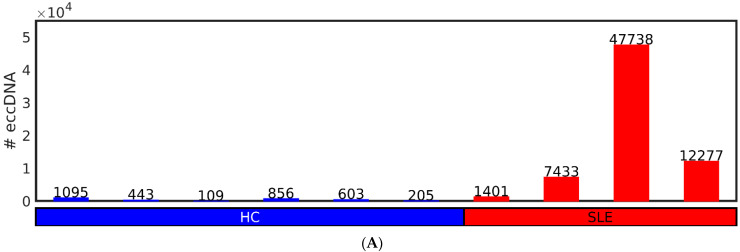

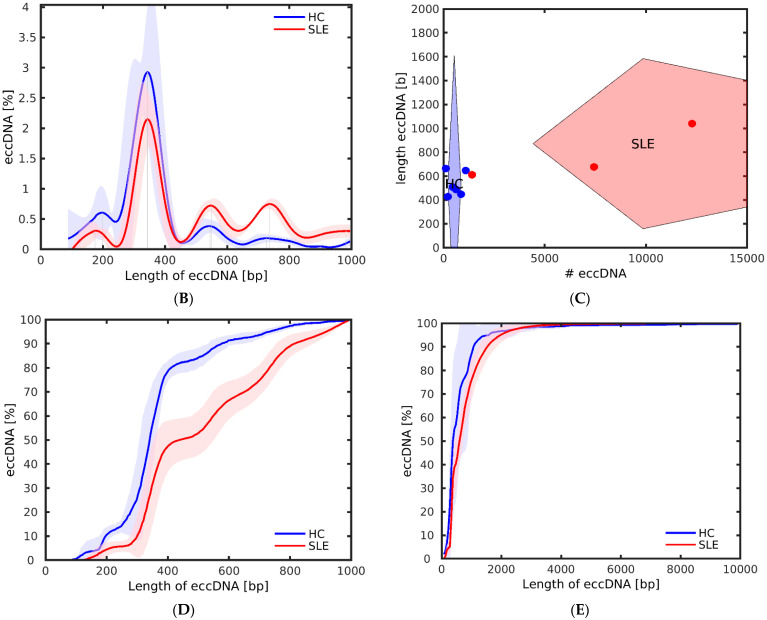
Distributions of number of unique sequence and length of cf-eccDNA in systemic lupus erythematosus (SLE) with DNASE1L3 deficiency and healthy control (HC). (**A**) Number (#) of unique eccDNAs in each sample of the SLE and HC groups up to a size of 10^4^ bp after merging and removal of eccDNAs with less than 2 split reads. (**B**) Periodic enrichment of eccDNA in the two groups in the size range from 0 to 10^3^ bp. The vertical lines mark the local maxima of the most abundant lengths after smoothing. (**C**) Rhomboid plot of the eccDNA lengths versus the number (#) of eccDNAs. The left, center, and right vertexes mark the µ – σ, µ, and µ + σ of the number of eccDNA length distribution in each of the of the groups of samples, respectively. The lower, center, and upper vertexes mark the µ − σ, µ, and µ + σ of the eccDNA length distribution, respectively. µ and σ are the mean and the standard deviation of the distributions, respectively. Each sample position in the length versus number eccDNA space is represented by a filled blue or red circle for the HC and SLE samples, respectively, with the exception of the SLE sample with 47,738 eccDNA that is located outside the map. Cumulative distribution of the lengths of the eccDNA in the range from (**D**) 0 to 10^3^ bp. (**E**) 0 to 10^4^ bp. The HC and SLE samples are depicted in blue and red, respectively. In the (**B**,**D**,**E**) panels, the shadowed blue and red regions cover the standard deviation of the enrichment across the HC and SLE groups, respectively.

We first checked for the differences in the number and length frequency distributions of the eccDNA in the SLE and HC groups. The cross-chromosomal load of unique eccDNA, up to a size of 10,000 bp, ranges from 109 to 1095 unique eccDNAs (µ ± sem = 552 ± 155) in HC, and 1401 to 47,738 eccDNAs (µ ± sem = 17,212 ± 10,416) in SLE (Figure 2A) (where µ is the mean and sem is the standard error of the mean). The Wilcoxon rank sum test indicates that the number of eccDNAs in HC is statistically significantly smaller than the number of eccDNAs in SLE with DNASE1L3 deficiency, at a 1% significance level with a *p*-value of 0.00476, i.e., showing a 0.0321-fold decrease in the eccDNA under the analyzed conditions.

### 3.2. Cf-eccDNA Length Distribution Is Distinct in SLE Compared to HC

The comparison of the smoothed length distribution profiles of SLE and HC shows lower peak SLE eccDNA clusters compared to the HC for lengths of approx. 200 bp and 350 bp, and a flip to higher peak SLE eccDNA clusters for lengths greater than 650 bp (Figure 2B). These results agree with the eccDNA size distribution analysis in Sin et al. [2]. Interestingly, lupus has been found to have a stronger mononucleosome length footprint than healthy controls, and even all of the studied cancer types in cfDNA [7]. Chan et al. [8] observed an increased frequency of long DNA molecules of above 250 bp and an increased amount of short plasma DNA molecules below 120 bp in lupus patients with DNASE1L3 disease-associated variants compared to healthy controls, but lower frequencies in the length range in-between. The rhomboid plots of the distributions of the lengths of the eccDNA versus their numbers of eccDNA show how the samples are separated between HC and SLE due to the higher numbers of eccDNA in SLE (Figure 2C). The cumulative eccDNA length curve shows the faster increase of longer eccDNA in SLE compared to HC after a length of 400 bp (Figure 2D). The majority of eccDNA has a length of less than 10,000 bp (Figure 2E). 

### 3.3. Differential Analysis Based on Split Reads Identifies Distinctive Genic cf-eccDNA Profile of SLE

**Figure 3:**
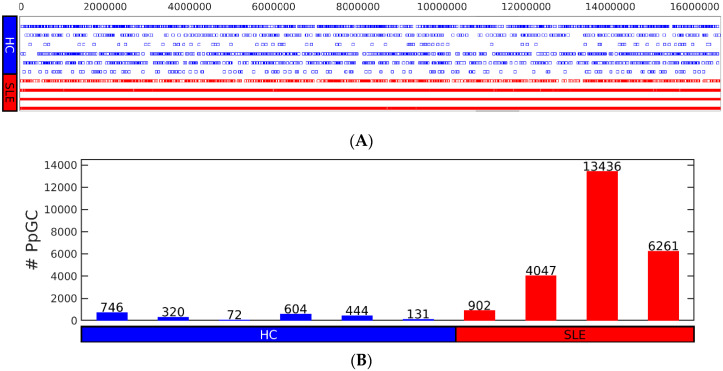

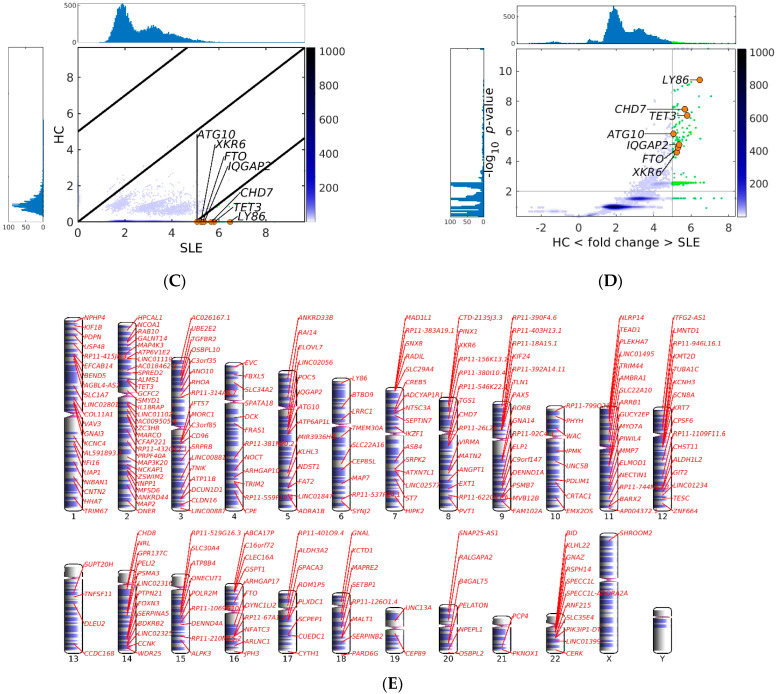
PpGCs-based sensitivity of circulomics to detect genic differences between systemic lupus erythematosus (SLE) with DNASE1L3 deficiency and healthy control (HC) in plasma. (**A**) Spy matrix of the produced per gene circles (PpGCs) detected in each sample. The columns represent the detected PpGCs shared by all the samples. Each square marks the detection of a PpGC in a sample. (**B**) Number (#) of PpGCs in each sample of the SLE and HC groups. (**C**) Pairwise scatter plot and (**D**) volcano plot of circulomics data from SLE and HC. Darker blue color corresponds to higher scattering density. The up-DPpGCs in the SLE samples are shown with green dots. Several gene positions are shown as orange circles. The levels are log2-scaled. The histograms visualize the PpGCs spectra. (**E**) Chromosomal landscaping of the up-DPpGCs in SLE compared to HC.

**Figure 4:**
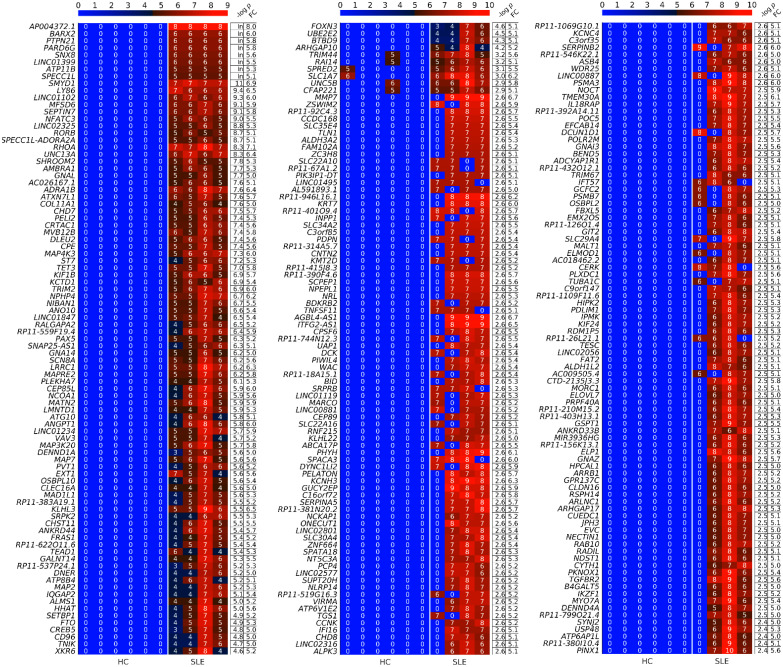
Heatmaps of the differentially up-produced per gene DNA circles (up-DPpGCs, 267) in the plasma of systemic lupus erythematosus (SLE) with DNASE1L3 deficiency compared to the healthy control (HC) group in decreasing order of significance. The color bar codifies the scaled split read count of the eccDNA per gene in a log2 scale. Higher count corresponds to a redder color. The –log10 (*p*-value) and the absolute value of the log2 of the fold change (FC) of the DPpGCs are presented in a table to the right of the heatmap. In: infinite.

**Figure 5:**
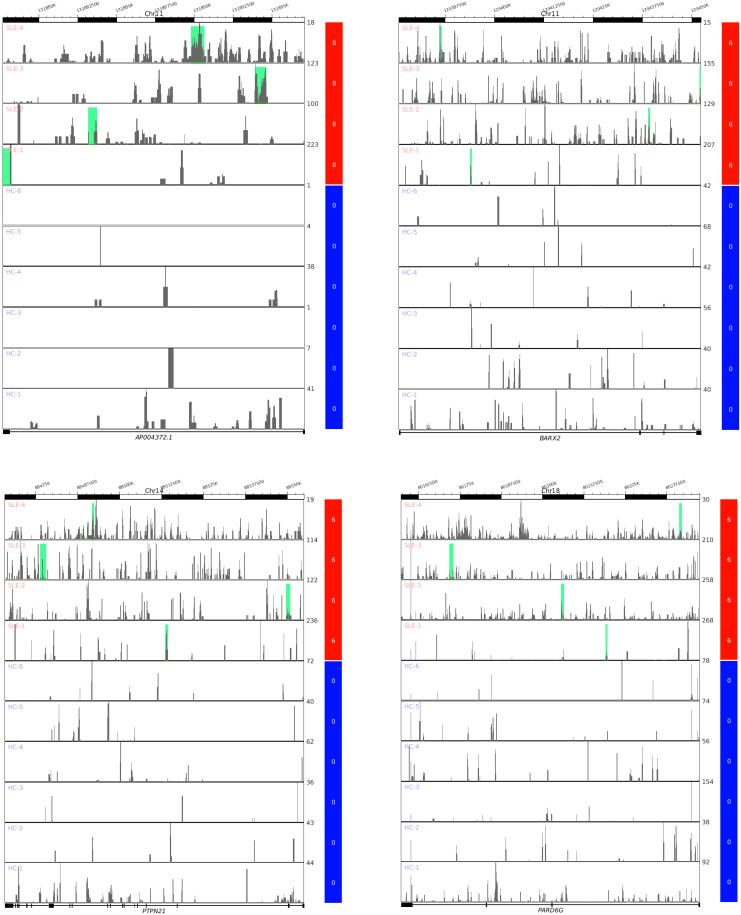
Track plots of the *loci* of the four top-ranked up-DPpGCs in systemic lupus erythematosus (SLE) with DNASE1L3 deficiency and healthy control (HC), and the corresponding gene coverage. Each horizontal line represents the length of a gene. The green bars represent the excision *loci* of the eccDNA. The color bars codify the scaled split read count of the eccDNA per gene in a log2 scale.

First, we checked the proportion of produced per gene circles (PpGCs) in the HC and SLE samples to confirm the possibility of performing differential analysis based on PpGCs (Figure 3) to detect differentially PpGCs (DPpGCs) between HC and SLE. Even though the cf-eccDNAs are less abundant in the HC samples, their dispersion is approximately equally distributed for each sample (Figure 3A). We observed a similar distribution of the number of PpGCs (Figure 3B) to that of the total number of unique cf-eccDNAs in all of the samples (Figure 2A). Additionally, the number of genic cf-eccDNAs (PpGC) constitutes a large proportion of the number of cf-eccDNAs for each sample. The pairwise scatter plot of SLE versus HC (Figure 3C) shows the existence of a difference in genic eccDNA between the two groups, and the volcano plot (Figure 3D) shows that this difference is statistically significant (significance level α_DPpGC_ = 0.01). We identified 267 statistically significant up-DPpGCs in the SLE group compared to the HC group, and no up-DPpGCs in the HC compared to SLE (Figure 3E and Figure 4). Noteworthy, the top 93 up-DPpGCs in the SLE are specific for SLE and are concurrently present in all SLE samples, but not in any of the HC samples (Figure 4). We performed a whole-gene length analysis and found that the eccDNA excised from the genes up-producing eccDNA in SLE are carrying only gene fragments and no complete genes. The *loci* of the top-ranked up-DPpGCs, together with the read coverage, are shown in the track plots in Figure 5 for the top four up-DPpGCs in SLE; the 93 specific up-DPpGCs present in all SLE samples are shown in Figure S1.

### 3.4. Gene Set Enrichment Analysis of up-DPpGCs in SLE Discloses Lupus Related Genes

**Figure 6:**
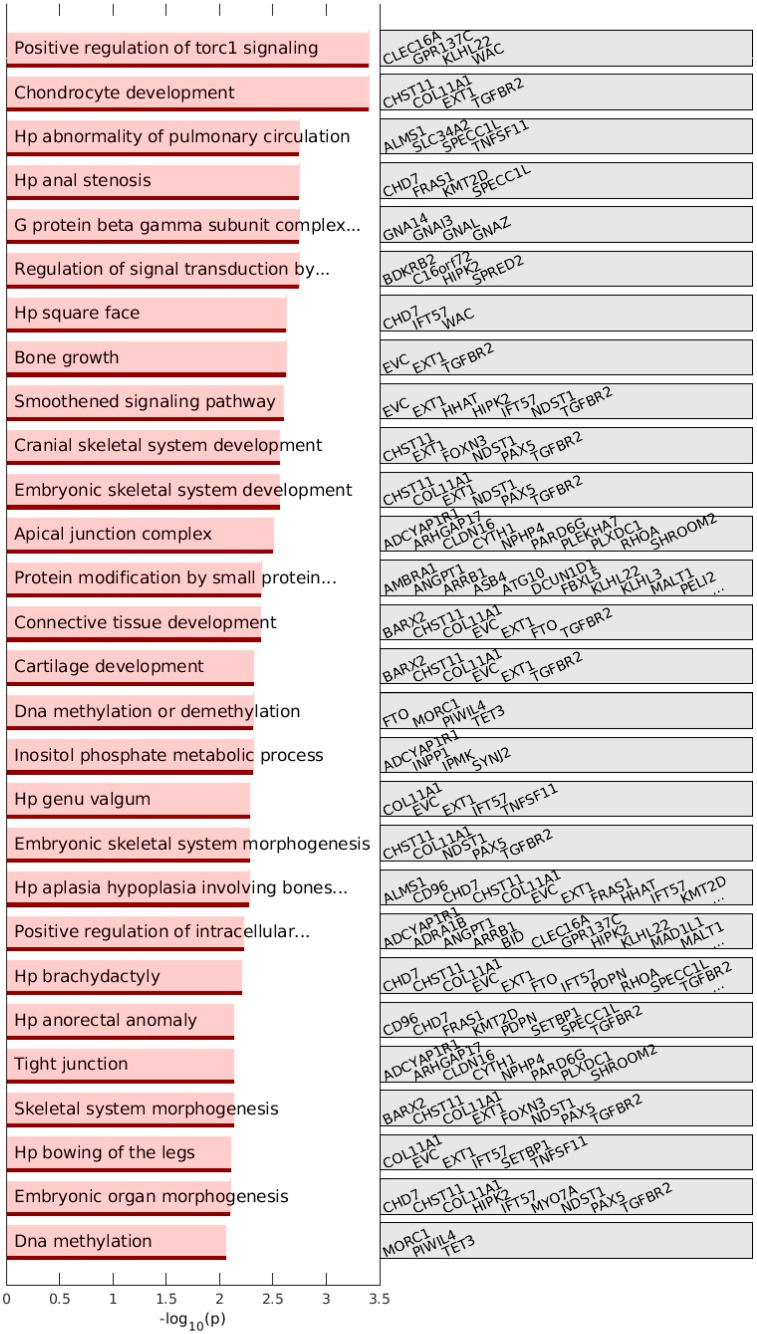
GSEA term enrichment analysis of DPpGCs. (Left) Bar plots of the –log10 (*p*-value) of the significantly enriched up-DPpGCs in SLE. (Right) List of genes in significant GSEA enrichment terms for the up-DPpGCs in SLE compared to HC. The number of genes in the signal set is 267. The number of genes in the background set is 1887. …: nonlisted genes.

We performed gene ontology (GO) enrichment analysis using the Gene Set Enrichment Analysis (GSEA) sets [9] for systematic functional association of the genes related to the up-DPpGCs. The top enriched ontology term of the up-DPpGCs in SLE is ‘positive regulation of torc1 signaling’ due to the detection of the up-DPpGCs in the genes *CLEC16A*, *GPR137C*, *KLHL22,* and *WAC* (Figure 6). The nutrient-responsive mammalian target of rapamycin TOR complex 1 (mTORC1) is a master regulator of cell growth, an important bridge between nutrition and metabolism, and impacts diverse processes involved in the promotion of autoimmunity in SLE [10,11]. It participates in the proliferation and differentiation of immune cells, affects the secretion of inflammatory cytokines, and alters cell autophagy levels. Upstream factors that drive increased mTOR activity in SLE are increased reactive oxygen intermediates (ROI) and nitric oxide, decreased levels of the reduced form of glutathione (GSH), and elevated mitochondrial potential. The mTOR pathway is involved in metabolic processes that predispose cells to die by necrosis and release oxidized DNA, which can have proinflammatory consequences [10]. Both upstream factors and mTORC1/2 are molecular targets mTOR inhibitors in SLE patients [11]. C-type lectin domain containing *16 A*, *CLEC16A*, is an immunity-associated gene that modulates thymic epithelial cell autophagy and alters T cell selection, with its silencing in mice protecting against autoimmunity [12]; its variants are associated with susceptibility to SLE [13], and it has reduced expression in peripheral leukocytes in SLE [14]. G protein-coupled receptor 137C, *GPR137C,* is an enzyme involved in the biosynthesis of gangliosides, functioning as antigens or receptors by recognizing lectins at the cell surface, and by modulating the charge density at the membrane surface. KLHL22 plays a conserved role to mediate the activation of mTORC1 and downstream events in mammals and nematodes, and has been found to promote tumorigenesis and aging through activating amino acid-dependent mTORC1 signalling [15]. WAC promotes mTORC1 activity by acting as an adaptor for the proper assembly of the TTT-Pontin/Reptin complex, as studied by David-Morrison et al. [16]. 

As SLE is an autoimmune disorder of the connective tissue, the other top GSEA term is ‘chondrocyte development’, represented by the genes *CHST11*, *COL11A*, *EXT1,* and *TGFBR2* (Figure 6). CHST11, which attaches sulfates to the 4-position of unsulfated chondroitin, the major component of cartilage, was found to be highly expressed in cutaneous lupus erythematosus [17]. Collagen alpha-1(XI) chain *COL11A* polymorphisms have been associated with SLE [18]. Exostosin 1, *EXT1,* is known to regulate chondrogenesis by modulation of WNT signaling [19]. EXT1 and EXT2 are the primary antigens for a subset of autoimmune diseases, including lupus [20]. They are primary antibodies for some types of membranous glomerulonephritis (MN) [21], with EXT1 positivity of kidney biopsies associated with better kidney function [22]. Transforming growth factor β receptor 2, TGFBR2, together with TGFBR1, binds TGFβ and regulates the transcription of genes related to immunosuppression [23].

Numerous other significant enriched GSEA terms are related to the skeletal system, e.g., ‘bone growth’, ‘embryonic skeletal system development’, ‘cranial skeletal system development’, ‘brachydactyly’, ‘bowing of the legs’, and terms related to the connective tissues are ‘connective tissue development’ and ‘cartilage development’. A member gene of some of the enriched terms related to cartilage and skeletal system morphogenesis is our top SLE up-DPpGCs gene *BARX2*. A SNP related to lupus nephritis (LN) has been found close to *BARX2* [24]. 

Another top and specific SLE up-DPpGCs gene that is part of the skeletal- and cartilage-related enriched GSEA terms is the transcription factor *PAX5*, a master regulator of B cell development and leukemogenesis [25]. Abnormalities in B cells play pivotal roles in the pathogenesis of SLE and LN, and dysregulation of B cell transcription factors, cytokines, and B cell–T cell interaction can result in aberrant B cell maturation and autoantibody production, with these immunological abnormalities leading to perturbations in circulating and infiltrating B cells in SLE and LN patients [26]. 

An intriguing GO biological process term among the top GSEA enrichment terms is ‘regulation of signal transduction by p53 class mediator’, represented by the genes *BDKRB2*, *C16orf72*, *HIPK2,* and *SPRED2*, where the bradykinin receptor B2, *BDKRB2,* is a G-protein coupled receptor involved in the regulation of cardiovascular and renal functions, as well as inflammation, and is a target for the tumor suppressor p53-mediated transcriptional activation [27]. *BdkrB2* inactivation results in the upregulation of checkpoint kinase 1 (Chk1) levels and potentiating phosphorylation of p53 on Ser23 by Chk1, an essential step in the pathway leading to renal dysgenesis [28]. Another member of this ontology is an unannotated gene, *C16ORF72*, renamed by Benslimane et al. [29] as *TAPR1* (telomere attrition and p53 response 1), who found that TAPR1 buffers against the deleterious consequences of telomere erosion or DNA damage by constraining p53. Activation of p53-mediated gene transcription is a critical cellular response to DNA damage, and involves a post-translational phosphorylation-acetylation cascade of p53. Puca et al. [30] uncovered a role for HIPK2 in activating p53 apoptotic transcription through regulating the balance between p53 acetylation and deacetylation, by stimulating on one hand corecruitment of p300 and p53Lys382 on apoptotic promoters, and on the other hand by inhibiting Sirt1 deacetylase activity. *HIPK2 is* elevated in rheumatic arthritis tissues, and inhibition of its expression inhibits chondrocyte apoptosis [31]. Additionally, among the enriched terms is ‘positive regulation of intracellular signal transduction’.

The list of enriched GSEA terms includes ‘dna methylation or demethylation’, represented by the *FTO*, *MORC1*, *PIWIL4,* and *TET3* genes (Figure 6). Multiple genetic variants associated with SLE have been found near and in TET3, pointing to the role of DNA demethylation in SLE [32]. Animal studies suggest that Tet2- and Tet3-mediated chromatin modification participated in the repression of CD86 on self-reactive B cells, a mechanism that may contribute to autoimmunity prevention. Tet2- and Tet3-deficient B cells led to hyperactivation of B cells, autoantibody production, and lupus-like disease in affected mice [33].

### 3.5. Harmonizome Enrichment Analysis of up-DPpGCs in SLE Discloses Autoimmune-Related Genes

SLE is the most common type of lupus. SLE is an autoimmune disease in which the immune system attacks its own tissues, causing widespread inflammation and tissue damage in the affected organs. It can affect the joints, skin, brain, lungs, kidneys, and blood vessels. The top functional terms of the Harmonizome [34] term enrichment analysis of the set of up-DPpGCs in SLE are ‘disease’ and ‘disease of anatomical entity’, followed by ‘lymphopenia’, ‘metabolic syndrome x’, ‘asthma’, ‘cardiovascular system disease’, ‘leukemia’, and ‘immune system disease’. The list of the genes associated with the up-DPpGCs of the more specific Harmonizome terms are presented in Table 3. Lymphopenia is a disorder of having an abnormally low level of lymphocytes that is frequent in SLE, and profound in 10% of cases; T lymphocytes, especially CD4+, are more affected than B cells; it is associated with disease activity, risk of flare, and damage scores, with a mostly bacterial infectious risk [35]. Metabolic syndrome (MetS) is a chronic inflammatory and prothrombotic state related to dysregulation of obesity-related adipokines, insulin resistance, and oxidative stress, while SLE is characterized by premature atherosclerosis that cannot be explained solely by traditional risk factors. MetS is more frequent in SLE patients and aggravates the risk of atherosclerotic vascular disease, diabetes mellitus, and chronic kidney disease. Since the elevation of proinflammatory cytokines is a common mechanism of SLE activity and obesity, MetS may be an important factor bridging chronic systemic inflammation and accelerated atherosclerosis in SLE. The role of adipokines, such as leptin and adiponectin, in SLE remains unclear, though high serum leptin and adiponectin levels are biomarkers of disease progression in SLE [36]. Another significant Harmonizome term was ‘asthma’. There is a significant association between asthma and increased risk of SLE [37]. Premature coronary heart disease is a major cause of morbidity and mortality in SLE, and is markedly increased in SLE patients compared with the general population, with epidemiologic and pathogenesis studies suggesting a great deal in common between the pathogenesis of SLE and that of atherosclerosis [38]. A combination of traditional risk factors such as hypertension and dyslipidemia, and nontraditional ones such as antiphospholipid antibodies, inflammation, and low antiphosphorylcholine, are implicated in the increased risk of cardiovascular disease in SLE [39]. Compared to the general population, people with SLE carry a greater risk of developing leukemia, and especially lymphoma [40].

### 3.6. Overlap between SLE up-DPpGCs and GWAS Catalogue

We checked for the intersection of the list of the SLE with DNASE1L3 deficiency up-DPpGCs and the gene variants in the GWAS catalog [41] of terms related with SLE and glomerulonephritis. For ‘childhood onset systemic lupus erythematosus’ (2 genes in GWAS) we found one overlap for *XKR6* [42]. For ‘systemic lupus erythematosus’ (449 genes in GWAS) we found 9 in our set of up-DPpGCs, namely, *CLEC16A*, *IL18RAP*, *LMNTD1*, *PINX1*, *PVT1*, *SLC1A7*, *SPRED2*, *TET3*, and a different one from the childhood onset risk locus in *XKR6* [43]. Additionally, for ‘response to cyclophosphamide in systemic lupus erythematosus with lupus nephritis’ (12 genes in GWAS), we found two overlap genes, *BARX2* and *HIPK2*. For ‘interferon alpha levels in systemic lupus erythematosus’ (5 genes in GWAS), there is one overlap gene, *ANKRD44,* found to function as a negative regulator in the type I IFN signaling pathway, with decreased expression of ANKRD44 constituting one of the mechanisms for the dysregulated IFN response in SLE [44]. The production of interferon-α (IFN-α) is a key feature of the innate immune system in SLE, with IFN-α levels increased in SLE patients during disease flares. For ‘acute glomerulonephritis’ (1 gene in GWAS) we found *OSBPL10* in our up-DPpGCs in SLE. We did not expand the check to autoimmune risk *loci*, though some of the up-DPpGCs are associated with such sites, e.g., *IKZF1* [43]. Another SLE up-DPpGC is *UNC13A,* a common risk *locus* for both amyotrophic lateral sclerosis and immune disorders [45].

### 3.7. Whole Genes in cf-eccDNA in SLE and HC

**Figure 7:**
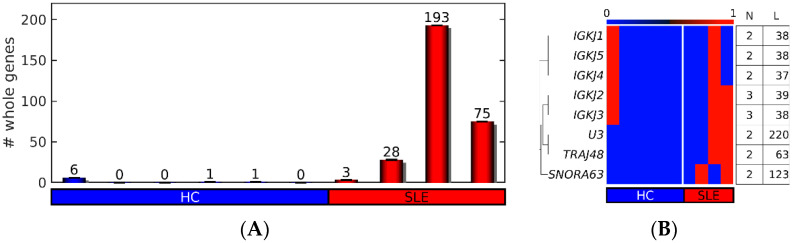
Whole genes carried by cf-eccDNA in SLE and HC. (**A**) Number (#) of whole genes per sample. (**B**) Whole genes in at least two samples. N: number of samples carrying the whole gene. L: length of the gene.

We found that the up-DPpGCs in SLE versus HC carry only gene fragments. However, we checked whether any cf-eccDNA in SLE and HC carries complete genes. We found that the cf-eccDNA from some samples, mainly from SLE, carry whole genes (Figure 7A). We checked the identity of these wholes genes for the recurrent cases of genes in at least two samples and we found that these correspond to short genes, mainly from the immune system, the T cell receptor α joining 48 (*TRAJ48*), and the five members of the immunoglobulin light κ chain family (*IGKJ*) (Figure 7B).

## 4. Discussion

We have identified a set of genes releasing specifically cf-eccDNA in SLE patients with DNASE1L3 deficiency caused by mutations. We showed that the differential analysis of the genic eccDNA, based on the split read signal of the eccDNA, confidently distinguishes SLE from HC, with functional associations of the identified eccDNA-producing genic sites. A large proportion of the cf-eccDNA in SLE and HC is generated by genic regions (Figure 2A and Figure 3B), and the genic information in the eccDNA in SLE and studies of the nucleases on cf-eccDNA has not been exploited so far [2]. The eccDNA profile of SLE with DNASE1L3 deficiency uncovered here is extremely specific compared to other genic eccDNA profiles identified so far in the skeletal muscle of sedentary and physically active aged men [3]. This is remarkable in the sense that here we analyzed circulomics data from plasma, and the eccDNA originates from different sites of the body. Additionally, enrichment of eccDNA is more difficult for plasma samples compared to tissue samples. One can argue that the clear eccDNA profile here in SLE is due to the DNASE1L3 deficiency and the better clearance of the eccDNA in the HC, though we have shown (Figure 3A) that the HC samples contain enough diverse genic eccDNA. We argue the idea of random release of eccDNA into the circulation with the demonstrated possibility of disease-related genic sites undergoing repair processes, and concordantly releasing eccDNA. Among the specific top up-DPpGCs in SLE with DNASE1L3 deficiency is the lymphocyte antigen 86, *LY86,* that is upregulated in prenephritic kidneys, and fluctuates with remission and progression towards relapse [46]. Interestingly, two of the patients (three SLE samples) had glomerulonephritis, but one, patient three, still had normal renal function. The up-DPpGCs carry gene fragments and, interestingly, circ-*IQGAP2* has been found to be a noninvasive biomarker of primary Sjögren’s syndrome, another rheumatic immune disorder [47]. *IQGAP2,* coding a protein required for the glomerular filtration barrier [48], is also specific and present in all samples of SLE up-DPpGC. These are just a few examples of how the SLE up-DPpGCs might not only reflect the actual pathology, but might also predict future disease progression. To confirm that, more samples and follow-up in time would be required. 

## 5. Conclusions

Cell-free extrachromosomal circular DNA (cf-eccDNA) is more stable than cell-free linear DNA that, analyzed in conjunction with our computational tool for detection of genes with differential abundance of eccDNA (DifCir) from circulomics data, shows that cf-eccDNA is a promising and possibly early biomarker in the autoimmune rheumatic diseases.

## Figures and Tables

**Table 1:** Patient characteristics based on the description provided in Sin et al. (2022).

	Patient 1	Patient 2	Patient 3
**Origin**	Albania	Italy	Italy
**Gender**	Male	Female	Male
**Diagnosis**	*At 5 years age:* steroid-resistant nephrotic syndrome. *At 7 years age:* membranous glomerulonephritis (stage II), ESRD	*At 4 years age:* Malar rash, diffuse urticarial erythematous rash, nonerosive arthritis, hemolytic anemia, hematuria, proteinuria, elevation of inflammation markers, increased creatinine, hypocomplementemia. *At 12 years age:* Class III C lupus nephritis. Despite treatment, progressed to ESRD	*At 3 years age:* Diffuse lymph node enlargement, recurrent fever episodes, increased inflammatory markers, arthritis, nonpruritic urticarial-like lesions, microcytic anemia, complement consumption. Fas-mediated apoptosis test, CD4− and CD8− lymphocyte counts, renal function and urine tests within normal ranges.
**ANA**		Positivity (1:640)	Borderline (1:80)
**Treatment**	*At 7 years age:* Kidney transplant, complicated by graft failure, and subsequent hemodialysis treatment	*At 12 years age:* Hemodialysis *At 13 years age:* Kidney transplant	
**Variations in *3DNASE1L3***	homozygous 97lfs*2 mutation of (c.290_291delCA/p.Thr97Ilefs*2)	heterozygous for c.290_291delCA (p.Thr97Ilefs*2) with a deletion in exon 5	homozygous 97lfs*2 mutation of (c.290_291delCA/p.Thr97Ilefs*2)

Abbreviations: (ANA) antinuclear antibody. (ESRD) end-stage renal disease.

**Table 2:** Total and mapped number (#) of reads to hg38 for each preprocessed data sample. HC, healthy control. SLE, systemic lupus erythematosus with DNASE1L3 deficiency.

# Reads	HC1	HC2-1	HC2-2	HC3-1	HC3-2	HC4	SLE-1	SLE-2	SLE-3	SLE-4
total	46,026,294	14,371,682	15,332,574	15,111,895	21,239,118	11,468,054	60,058,303	141,643,825	186,738,575	267,286,121
duplicates	32,565,823	9,220,429	7,134,077	11,224,979	12,724,471	7,093,619	43,427,190	118,546,354	160,046,604	244,961,765
mapped	41,530,639	11,212,079	8,373,237	13,397,961	14,779,795	8,077,581	46,065,504	13,123,3504	176,363,140	266,346,580
%mapped	90.23	78.02	54.61	88.66	69.59	70.44	76.7	92.65	94.44	99.65

**Table 3:** List of genes in significant Harmonizome enrichment terms for the up-DPpGCs in SLE with DNASE1L3 deficiency compared to HC. The number of genes in the signal set is 267. The number of genes in the background set is 1887.

Id Term	Genes
0000047 lymphopenia	*ALDH1L2*, *ALDH3A2*, *ATG10*, *CEP89*, *DLEU2*, *EXT1*, *GALNT14*, *IFT57*, *INPP1*, *IQGAP2*, *KIF24*, *NCOA1*, *PHYH*, *PVT1*, *RAB10*, *SPRED2*, *SRPK2*, *TNFSF11*, *TRIM44*, *USP48*
0000163 metabolic syndrome x	*ARHGAP10*, *CRTAC1*, *DNER*, *FTO*, *GIT2*, *ZNF664*
0000050 asthma	*ADRA1B*, *ALDH1L2*, *ALDH3A2*, *ANGPT1*, *ATG10*, *ATXN7L1*, *BARX2*, *BEND5*, *CEP89*, *CHD7*, *CREB5*, *DLEU2*, *DNER*, *EFCAB14*, *EXT1*, *FOXN3*, *GALNT14*, *GNA14*, *IFT57*, *IL18RAP*, *INPP1*, *IQGAP2*, *KIF24*, *NCOA1*, *ONECUT1*, *PHYH*, *PINX1*, *PVT1*, *RAB10*, *SPRED2*, *SRPK2*, *TLN1*, *TNFSF11*, *TRIM44*, *USP48*, *WDR25*
0000070 cardiovascular system disease	*ALDH1L2*, *ALDH3A2*, *ANGPT1*, *ANKRD33B*, *ANKRD44*, *ANO10*, *ARHGAP10, ARRB1, ATG10*, *ATP8B4*, *ATXN7L1*, *BARX2*, *BID*, *BTBD9*, *CD96*, *CEP85L*, *CEP89*, *CFAP221*, *CHST11*, *CLDN16*, *CLEC16A*, *CNTN2*, *CPE*, *CREB5*, *CRTAC1*, *CUEDC1*, *DENND1A*, *DENND4A*, *DLEU2*, *DNER*, *ELMOD1*, *ELOVL7*, *EMX2OS*, *EXT1*, *FAT2*, *FOXN3*, *FRAS1*, *FTO*, *GALNT14*, *GIT2*, *GNA14*, *GNAL*, *GPR137C*, *HHAT*, *HPCAL1*, *IFI16*, *IFT57*, *IL18RAP*, *INPP1*, *IPMK*, *IQGAP2*, *JPH3*, *KCNH3*, *KCTD1*, *KIF1B*, *KIF24*, *KLHL22*, *KLHL3*, *LMNTD1*, *LY86*, *MAD1L1*, *MAP2*, *MAP4K3*, *MAP7*, *MAPRE2*, *MARCO*, *MATN2*, *MORC1*, *MYO7A*, *NCOA1*, *NDST1*, *NFATC3*, *OSBPL10*, *PARD6G*, *PAX5*, *PCP4*, *PHYH*, *PINX1*, *PKNOX1*, *PLEKHA7*, *PLXDC1*, *POC5*, *PVT1*, *RAB10*, *RAI14*, *RALGAPA2*, *RORB*, *SETBP1*, *SHROOM2*, *SLC30A4*, *SPACA3*, *SPECC1L*, *SPRED2*, *SRPK2*, *SRPRB*, *SYNJ2*, *TEAD1*, *TET3*, *TGFBR2*, *TNFSF11*, *TNIK*, *TRIM44*, *TRIM67*, *UNC5B*, *USP48*, *VAV3*, *WAC*, *XKR6*, *ZNF664*
0000043 leukemia	*ALDH1L2*, *ALDH3A2*, *ALPK3*, *ANKRD44*, *ATG10*, *ATP8B4*, *CEP89*, *CHST11*, *DENND1A*, *DLEU2*, *DNER*, *EXT1*, *FTO*, *GALNT14*, *GNAL*, *GNAZ*, *IFT57*, *IKZF1*, *INPP1*, *IQGAP2*, *KIF24*, *MAD1L1*, *MAP2*, *MYO7A*, *NCOA1*, *NLRP14*, *PHYH*, *PVT1*, *RAB10*, *SCN8A*, *SPRED2*, *SRPK2*, *TNFSF11*, *TRIM44*, *USP48*
0000057 immune system disease	*ADRA1B*, *ALDH1L2*, *ALDH3A2*, *ALPK3*, *ANGPT1*, *ANKRD44*, *ANO10*, *ARHGAP17*, *ATG10*, *ATP6V1E2*, *BARX2*, *BID*, *BTBD9*, *CEP89*, *CLEC16A*, *CNTN2*, *CREB5*, *CRTAC1*, *DCUN1D1*, *DENND1A*, *DENND4A*, *DLEU2*, *DNER*, *EXT1*, *FOXN3*, *GALNT14*, *GNA14*, *GNAL*, *GPR137C*, *GUCY2EP*, *HPCAL1*, *IFI16*, *IFT57*, *IKZF1*, *IL18RAP*, *INPP1*, *IQGAP2*, *KIF1B*, *KIF24*, *MAD1L1*, *MAP2*, *MAP7*, *MATN2*, *NCOA1*, *NFATC3*, *OSBPL10*, *PAX5*, *PCP4*, *PDPN*, *PHYH*, *PLEKHA7*, *PVT1*, *RAB10*, *RALGAPA2*, *SETBP1*, *SMYD1*, *SPRED2*, *SRPK2*, *SUPT20H*, *TNFSF11*, *TNIK*, *TRIM44*, *UBE2E2*, *UNC13A*, *USP48*, *VAV3*, *KR6*

## Data Availability

This study makes use of data generated by The Chinese University of Hong Kong (CUHK) Circulating Nucleic Acids Research Group, as reported by Sin et al. in JCI insight (doi: 10.1172/jci.insight.156070). The data have been deposited at the European Genome-Phenome Archive (https://ega-archive.org/datasets) hosted by the European Bioinformatics Institute (accession EGAS00001005873.)

## Supplementary Materials

The following supporting information can be downloaded at: https://www.mdpi.com/article/10.3390/cells12071061/s1, Figure S1: Track plots of the excision *loci* of the 96 top-ranked up-DPpGCs in systemic lupus erythematosus (SLE) with DNASE1L3 deficiency and healthy control (HC), and the corresponding gene coverage.

## Abbreviations

cfDNA	Cell-free DNA
cf-eccDNA	Cell-free eccDNA
CPpGCs	Common produced per gene DNA circles
DifCir	Method and tool for differential analysis of circular DNA
DPpGCs	Differentially produced per gene DNA circles
eccDNA	Extrachromosomal circular DNA
FC	Fold change
GO	Gene ontology
GSEA	Gene set enrichment analysis
LN	Lupus nephritis
PpGCs	Produced per gene DNA circles
sem	Standard error of the mean
SLE	Systemic lupus erythematosus
SNP	Single nucleotide polymorphism
µ	Mean
σ	Standard deviation
