# Supplementary material for: Systemic Lupus Erythematosus Patients with DNASE1L3·Deficiency Have a Distinctive and Specific Genic Circular DNA Profile in Plasma
Source: Cells. 2023 Mar 31;12(7):1061. doi: 10.3390/cells12071061 (PMC10093232; doi:10.3390/cells12071061)
Supplement: Supplementary file 1 [file cells-12-01061-s001.zip › cells-2259055-supplementary.pdf]

Systemic lupus erythematosus patients with DNASE1L3·deficiency have a distinctive and specific genic circular DNA profile in plasma

Daniela Gerovska[1,*] and Marcos J. Araúzo-Bravo[1,2,3,4,5,*]

Special Issue "Systemic Lupus Erythematosus: Update of Pathogenesis and Target Therapy"

Daniela Gerovska[1*] and Marcos J. Araúzo-Bravo[1,2,3,4,5*]

[1] Computational Biology and Systems Biomedicine, Biodonostia Health Research Institute, Calle Doctor Begiristain s/n, 20014, San Sebastian, Spain
[2] Basque Foundation for Science, IKERBASQUE, Calle María Díaz Harokoa 3, 48013, Bilbao, Spain
[3] CIBER of Frailty and Healthy Aging (CIBERfes), Madrid, Spain
[4] Max Planck Institute for Molecular Biomedicine, Computational Biology and Bioinformatics, Röntgenstr. 20, 48149, Münster, Germany
[5] Department of Cell Biology and Histology, Faculty of Medicine and Nursing, University of Basque Country (UPV/EHU), 48940 Leioa, Spain

* Corresponding authors: Daniela Gerovska: daniela.gerovska@biodonostia.org, Marcos J. Araúzo-Bravo: mararabra@yahoo.co.uk

Daniela Gerovska ORCID: orcid.org/0000-0003-0671-4277
Marcos J. Araúzo-Bravo ORCID: orcid.org/0000-0002-3264-464X

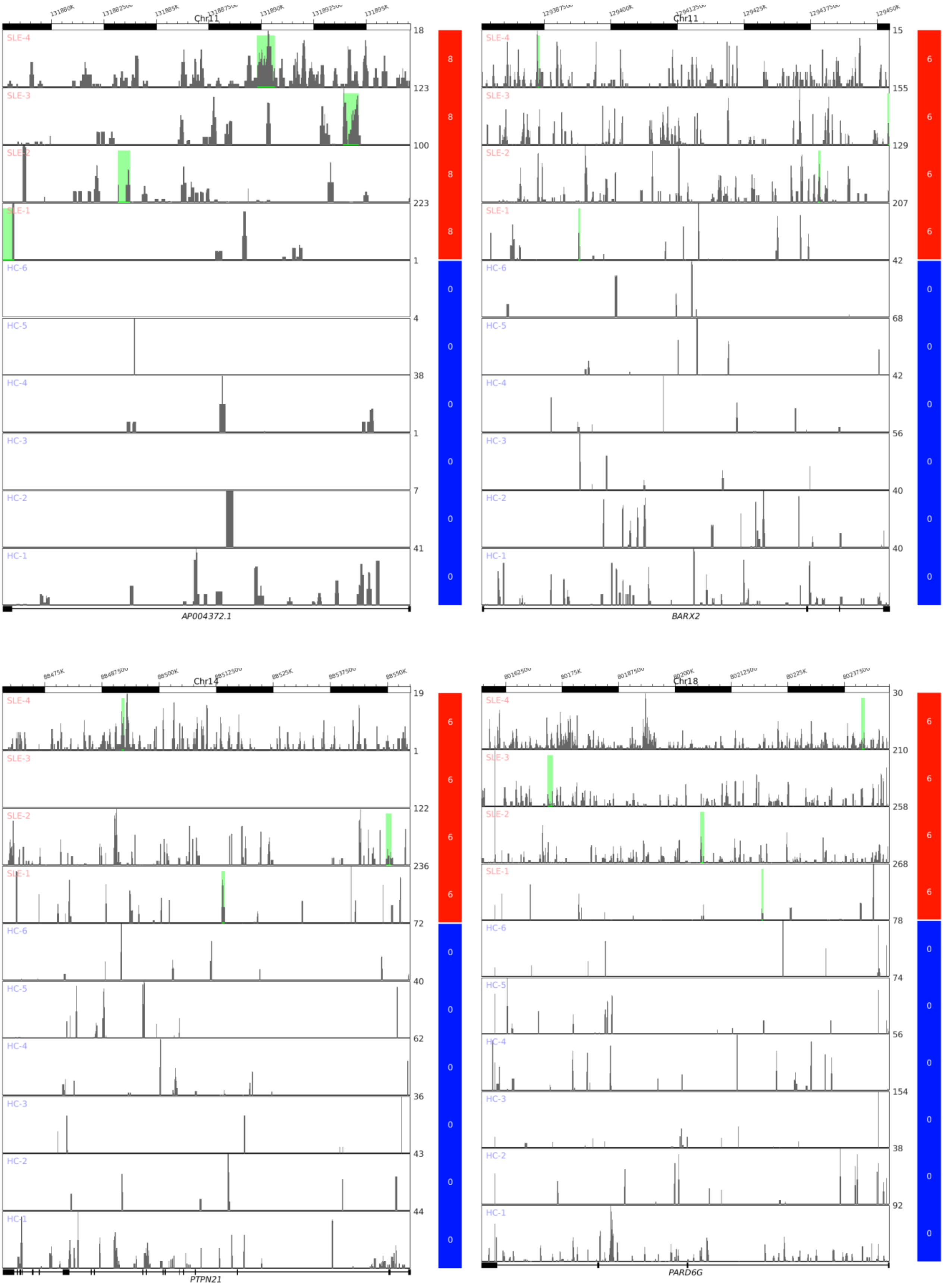

Chr11
AP004372.1
BARX2
Chr14
PTPN21
Chr18
PARD6G
SLE-4
SLE-3
SLE-2
SLE-1
HC-6
HC-5
HC-4
HC-3
HC-2
HC-1

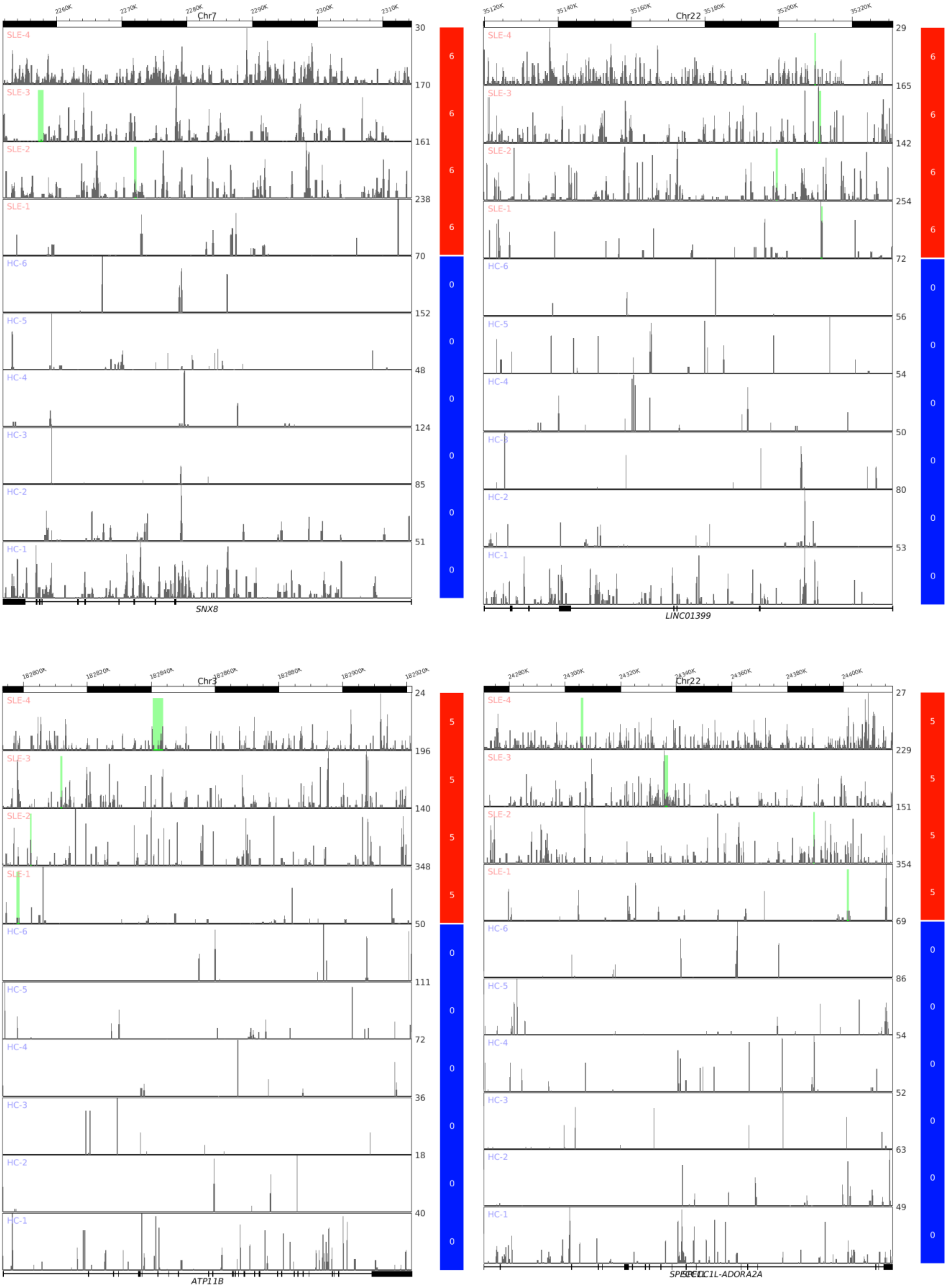
Chr7
2260K
2270K
2280K
2290K
2300K
2310K
SLE-4
SLE-3
SLE-2
SLE-1
HC-6
HC-5
HC-4
HC-3
HC-2
HC-1
30
170
161
238
70
152
48
124
85
51
6
6
6
6
0
0
0
0
0
0
SNX8
Chr22
35120K
35140K
35160K
35180K
35200K
35220K
29
165
142
254
72
56
54
50
80
53
LINC01399
Chr3
182800K
182820K
182840K
182860K
182880K
182900K
182920K
24
196
140
348
50
111
72
36
18
40
5
5
5
5
ATP11B
Chr22
24280K
24300K
24320K
24340K
24360K
24380K
24400K
27
229
151
354
69
86
54
52
63
49
SPECC1L-ADORA2A

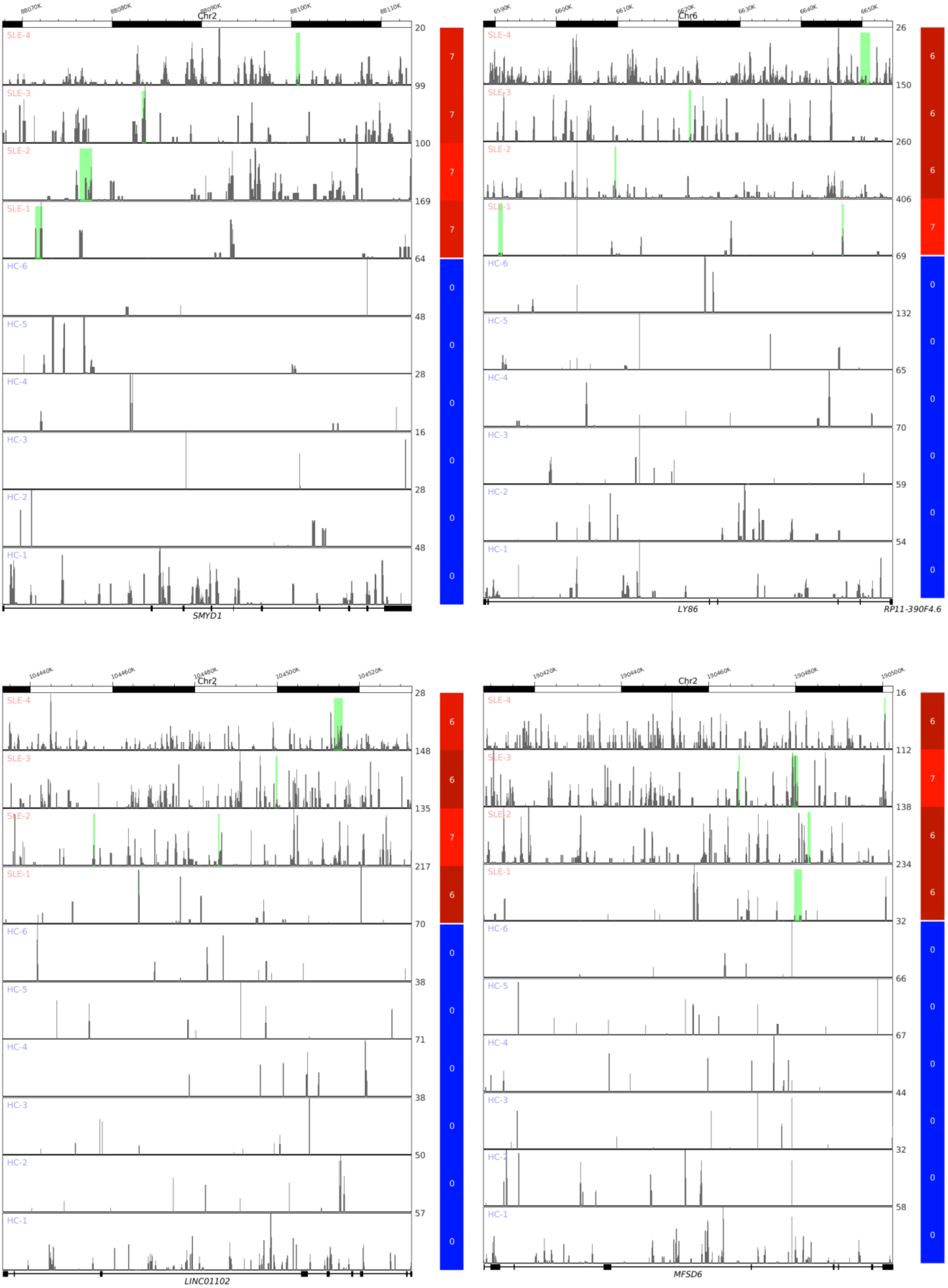
Chr2
SLE-4
SLE-3
SLE-2
SLE-1
HC-6
HC-5
HC-4
HC-3
HC-2
HC-1
SMYD1
Chr6
LY86
RP11-390F4.6
Chr2
LINC01102
Chr2
MFSD6

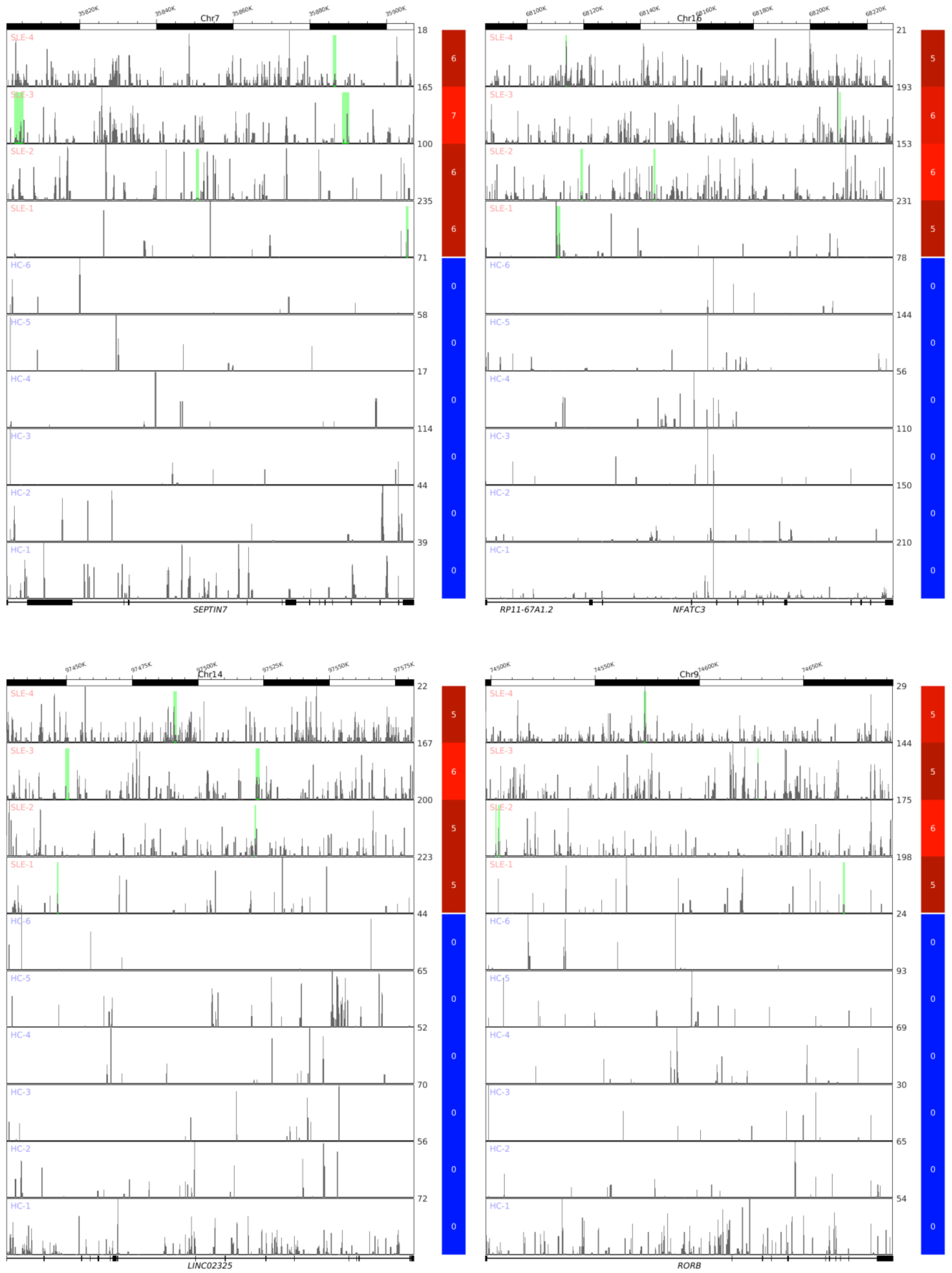

Chr7
35820K
35840K
35860K
35880K
35900K
SLE-4
SLE-3
SLE-2
SLE-1
HC-6
HC-5
HC-4
HC-3
HC-2
HC-1
SEPTIN7
Chr16
68100K
68120K
68140K
68160K
68180K
68200K
68220K
RP11-67A1.2
NFATC3
Chr14
97450K
97475K
97500K
97525K
97550K
97575K
LINC02325
Chr9
74500K
74550K
74600K
74650K
RORB

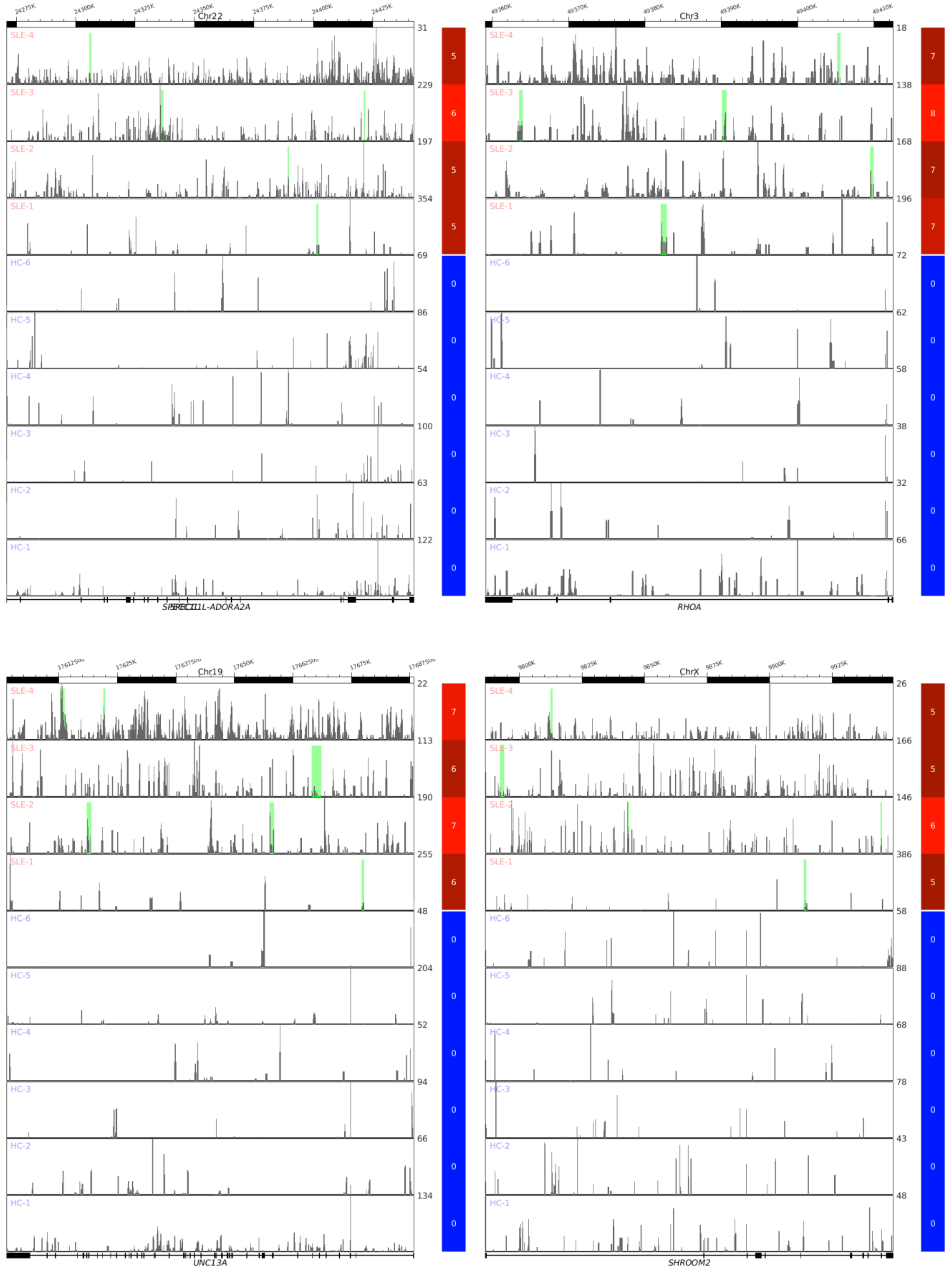

Chr22
SPECC1L-ADORA2A
Chr3
RHOA
Chr19
UNC13A
ChrX
SHROOM2
SLE-4
SLE-3
SLE-2
SLE-1
HC-6
HC-5
HC-4
HC-3
HC-2
HC-1

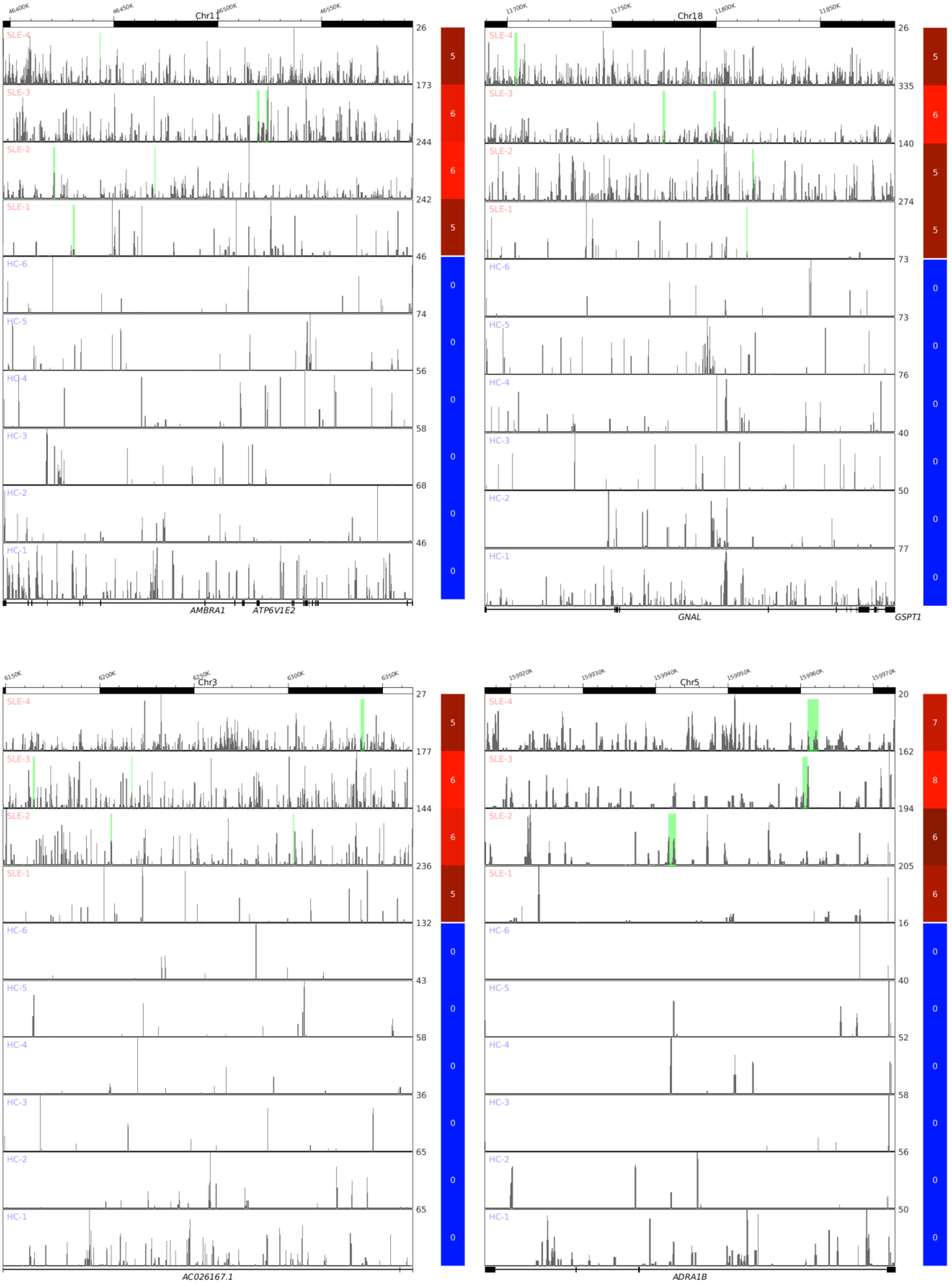
Chr11
SLE-4
SLE-3
SLE-2
SLE-1
HC-6
HC-5
HC-4
HC-3
HC-2
HC-1
AMBRA1
ATP6V1E2
Chr18
GNAL
GSPT1
Chr3
AC026167.1
Chr5
ADRA1B

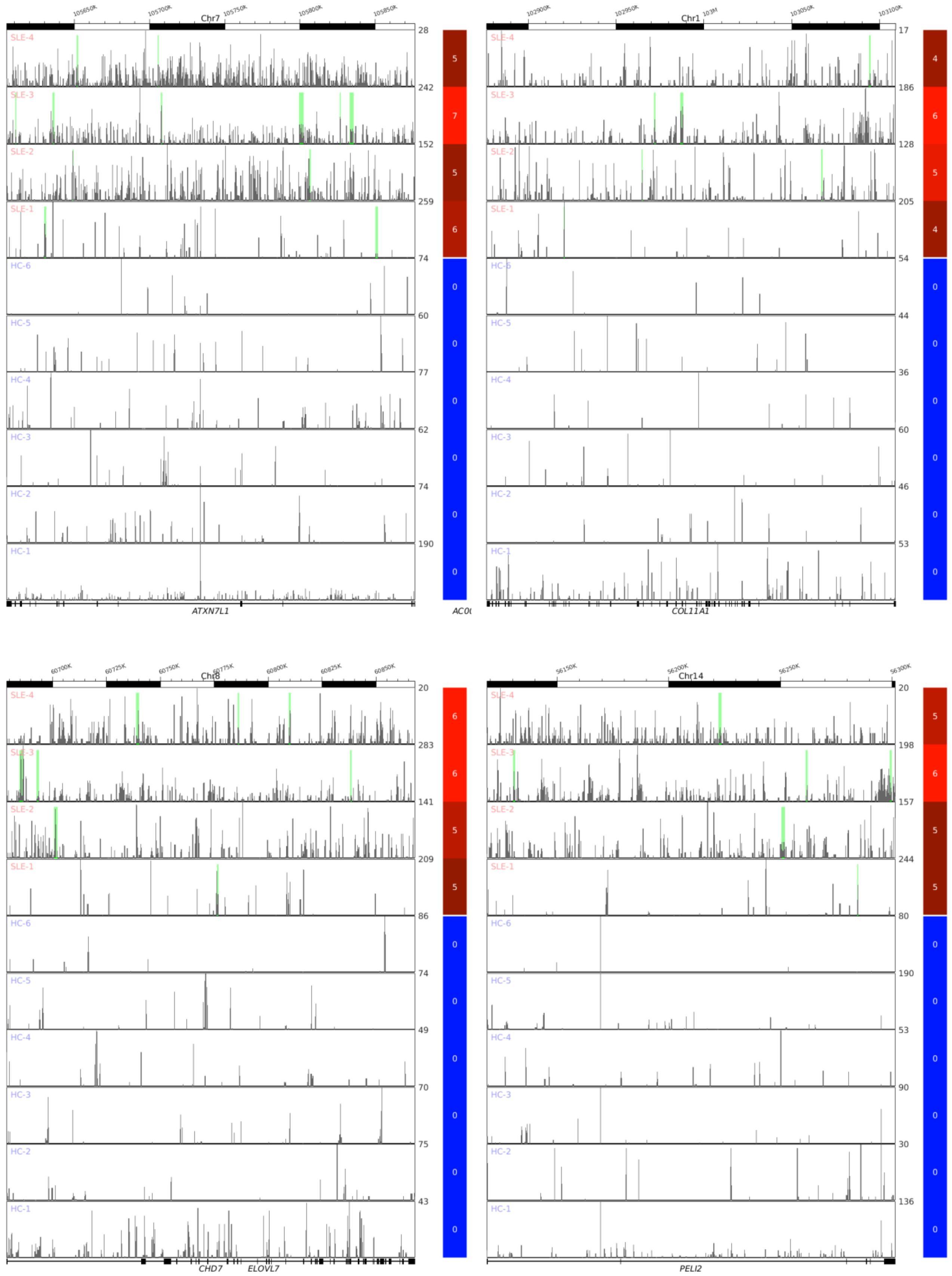

Chr7
105650K
105700K
105750K
105800K
105850K
Chr1
102900K
102950K
103M
103050K
103100K
Chr8
60700K
60725K
60750K
60775K
60800K
60825K
60850K
Chr14
56150K
56200K
56250K
56300K
SLE-4
SLE-3
SLE-2
SLE-1
HC-6
HC-5
HC-4
HC-3
HC-2
HC-1
ATXN7L1
COL11A1
CHD7
ELOVL7
PELI2

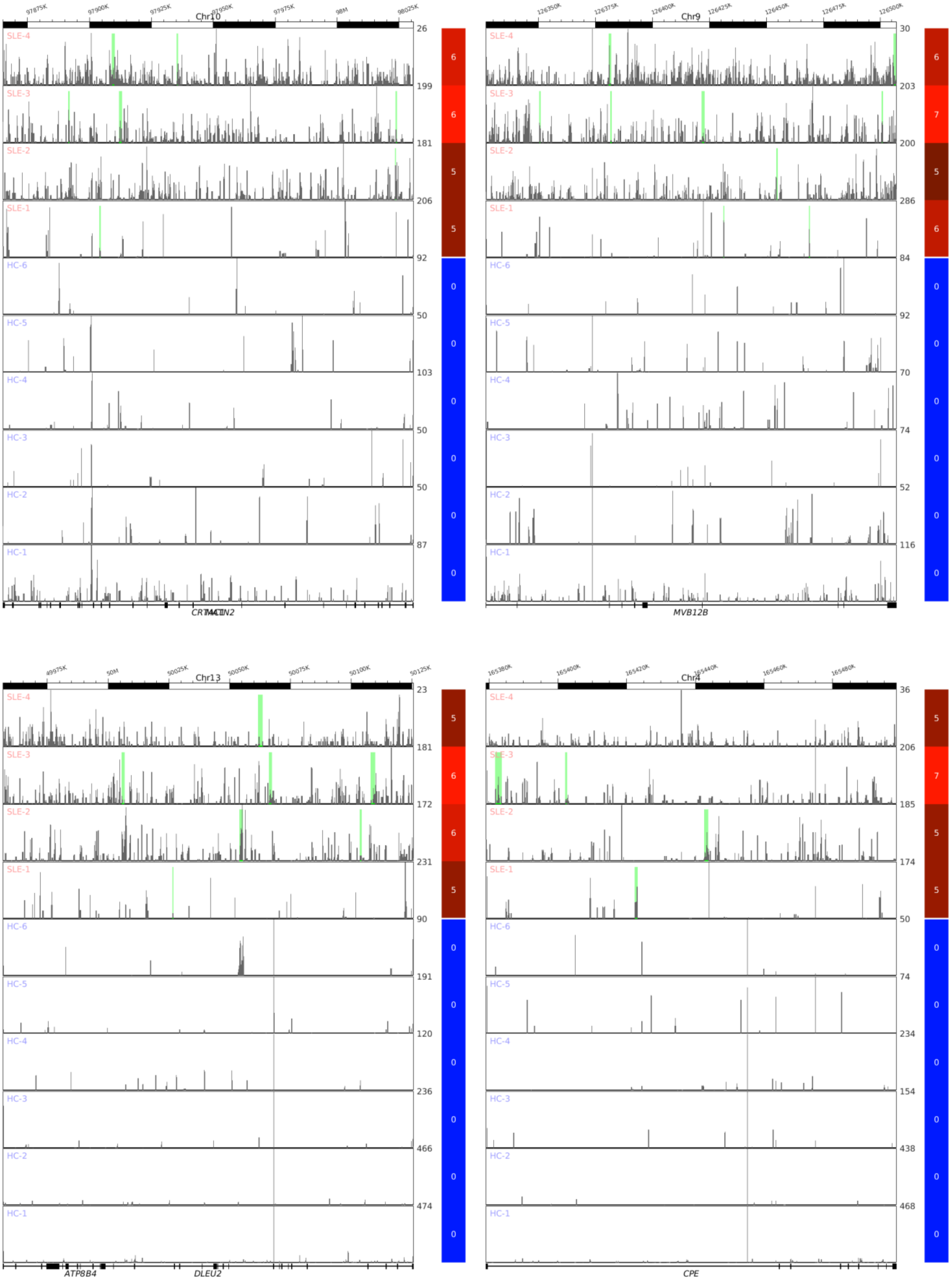
Chr10
Chr9
Chr13
Chr4
SLE-4
SLE-3
SLE-2
SLE-1
HC-6
HC-5
HC-4
HC-3
HC-2
HC-1
MVB12B
ATP8B4
DLEU2
CPE

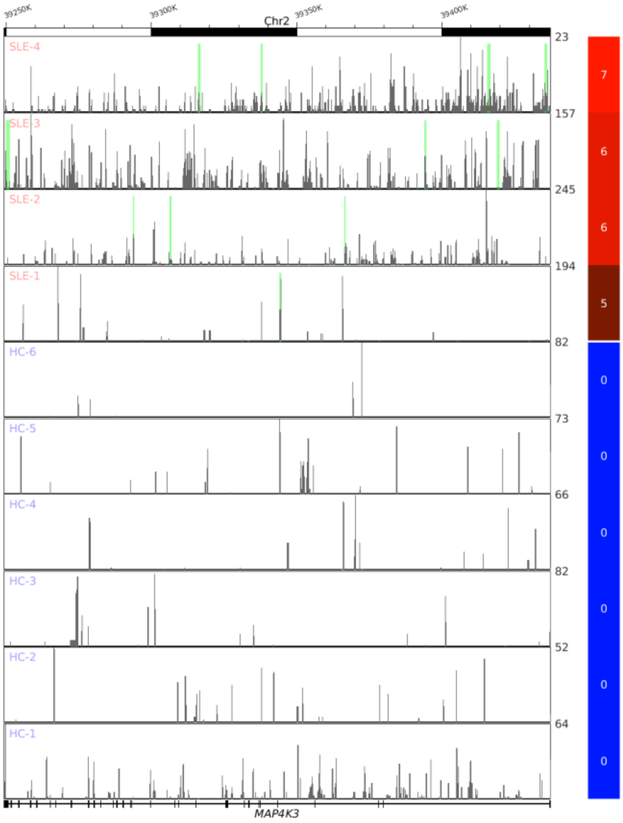

Chr2
39250K
39300K
39350K
39400K
SLE-4
SLE-3
SLE-2
SLE-1
HC-6
HC-5
HC-4
HC-3
HC-2
HC-1
MAP4K3

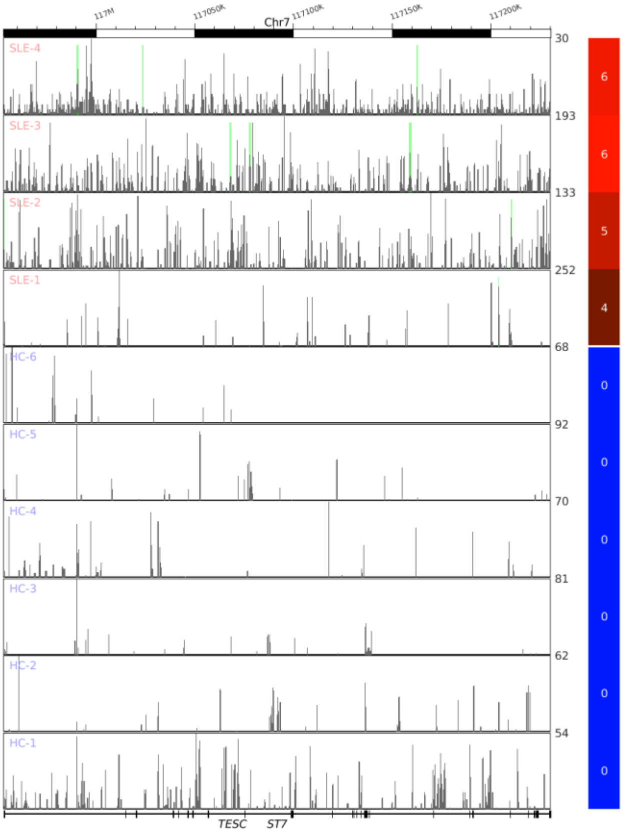

Chr7
117M
117050K
117100K
117150K
117200K
SLE-4
SLE-3
SLE-2
SLE-1
HC-6
HC-5
HC-4
HC-3
HC-2
HC-1
TESC
ST7

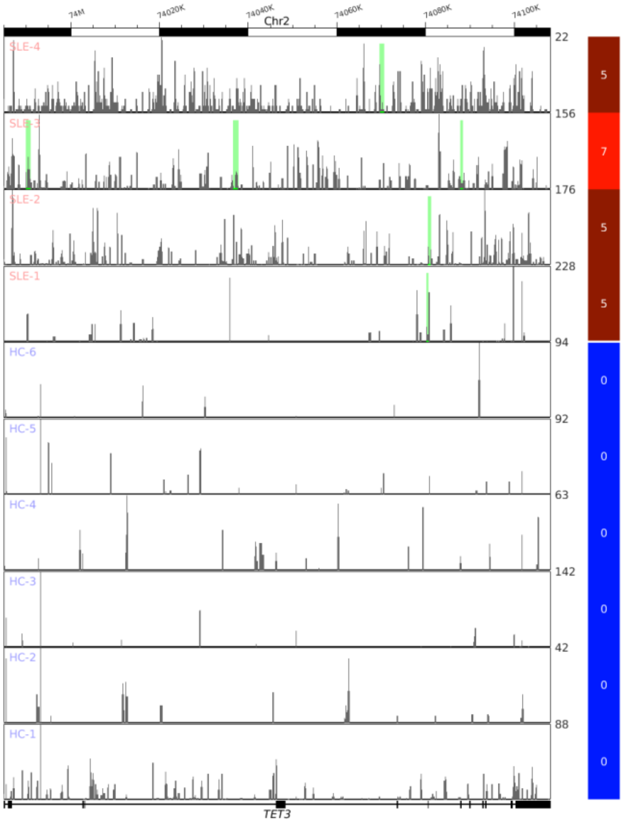

Chr2
74M
74020K
74040K
74060K
74080K
74100K
SLE-4
SLE-3
SLE-2
SLE-1
HC-6
HC-5
HC-4
HC-3
HC-2
HC-1
TET3

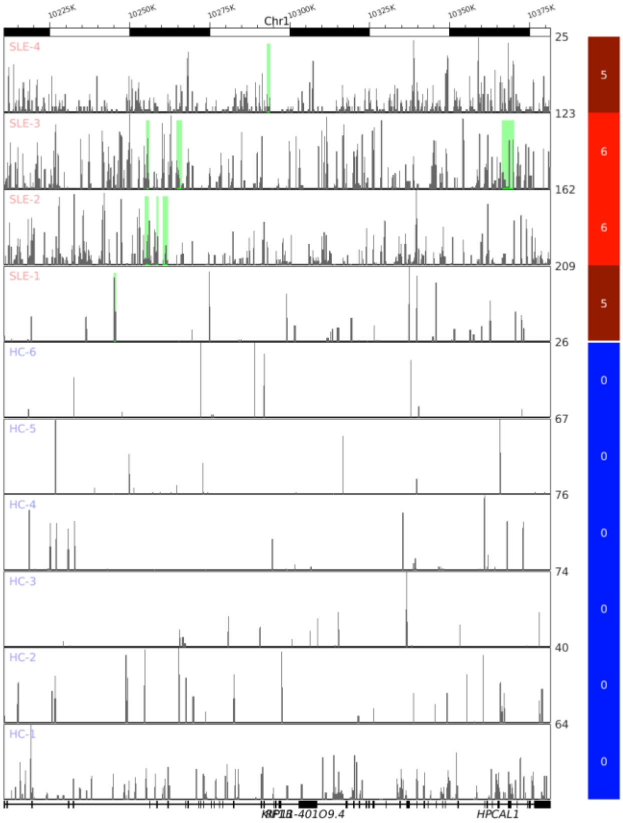

Chr1
10225K
10250K
10275K
10300K
10325K
10350K
10375K
SLE-4
SLE-3
SLE-2
SLE-1
HC-6
HC-5
HC-4
HC-3
HC-2
HC-1
HPCAL1

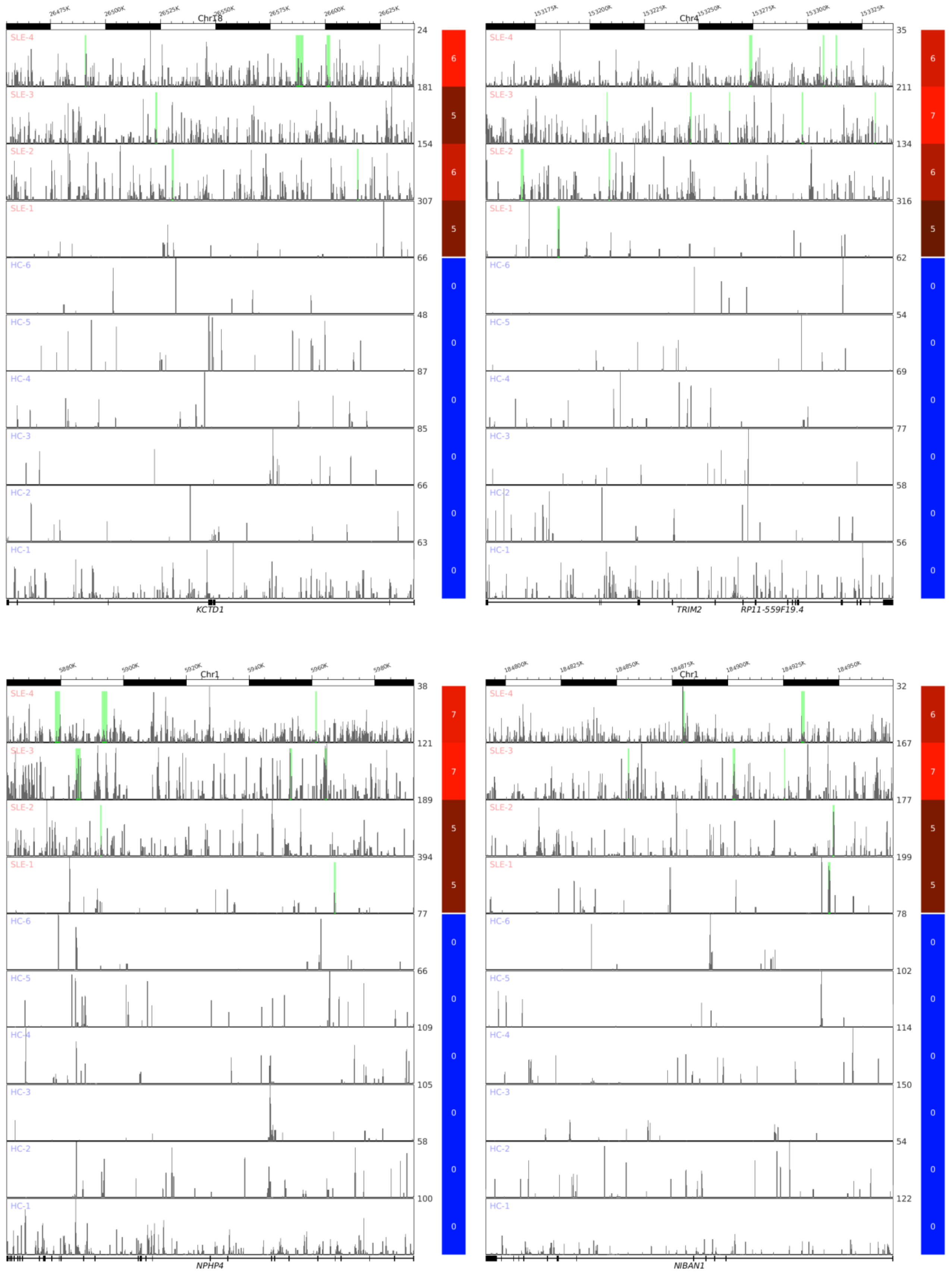
Chr18
SLE-4
SLE-3
SLE-2
SLE-1
HC-6
HC-5
HC-4
HC-3
HC-2
HC-1
KCTD1
Chr4
TRIM2
RP11-559F19.4
Chr1
NPHP4
Chr1
NIBAN1

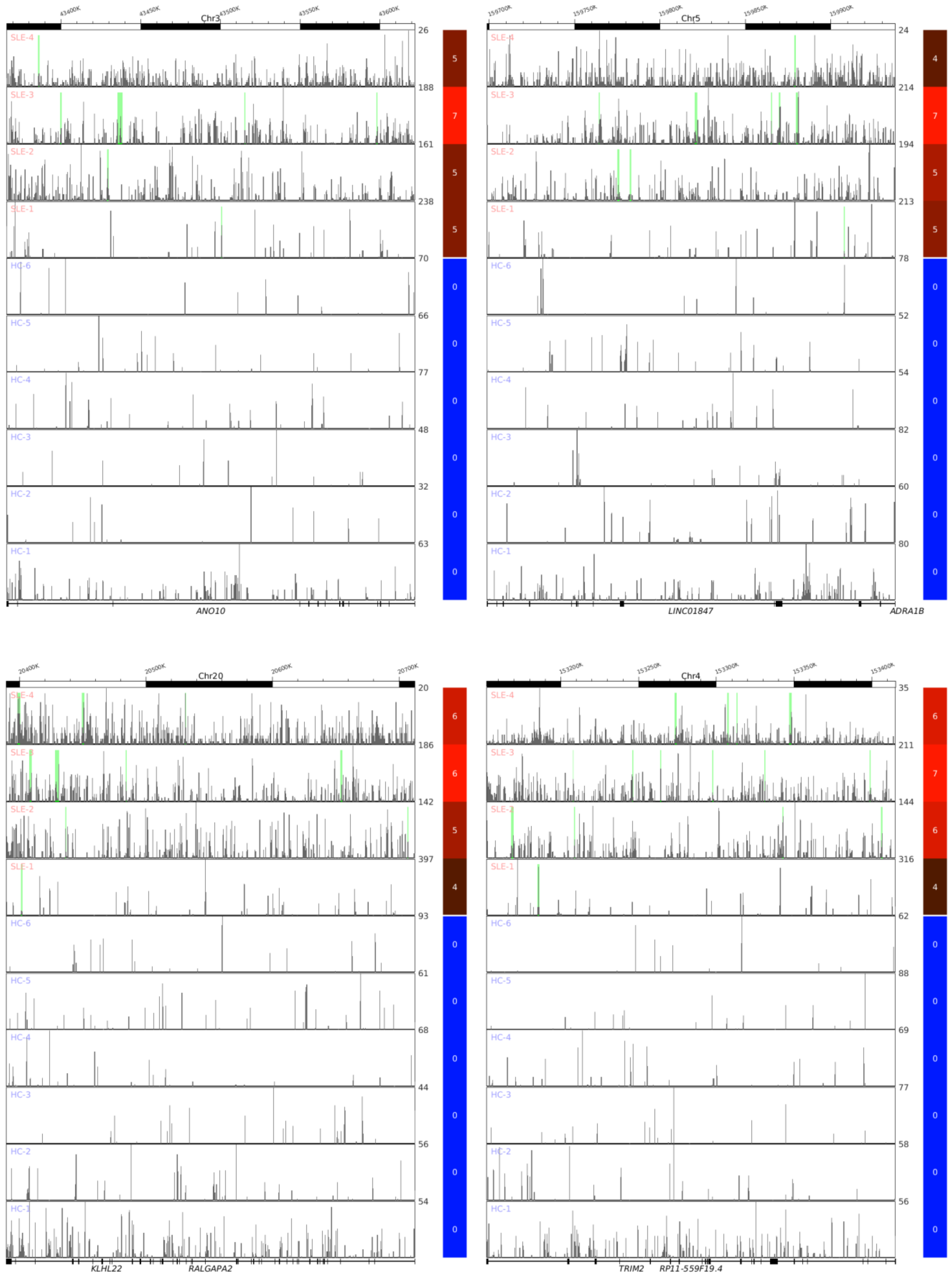
Chr3
SLE-4
SLE-3
SLE-2
SLE-1
HC-6
HC-5
HC-4
HC-3
HC-2
HC-1
ANO10
Chr5
LINC01847
ADRA1B
Chr20
KLHL22
RALGAPA2
Chr4
TRIM2
RP11-559F19.4

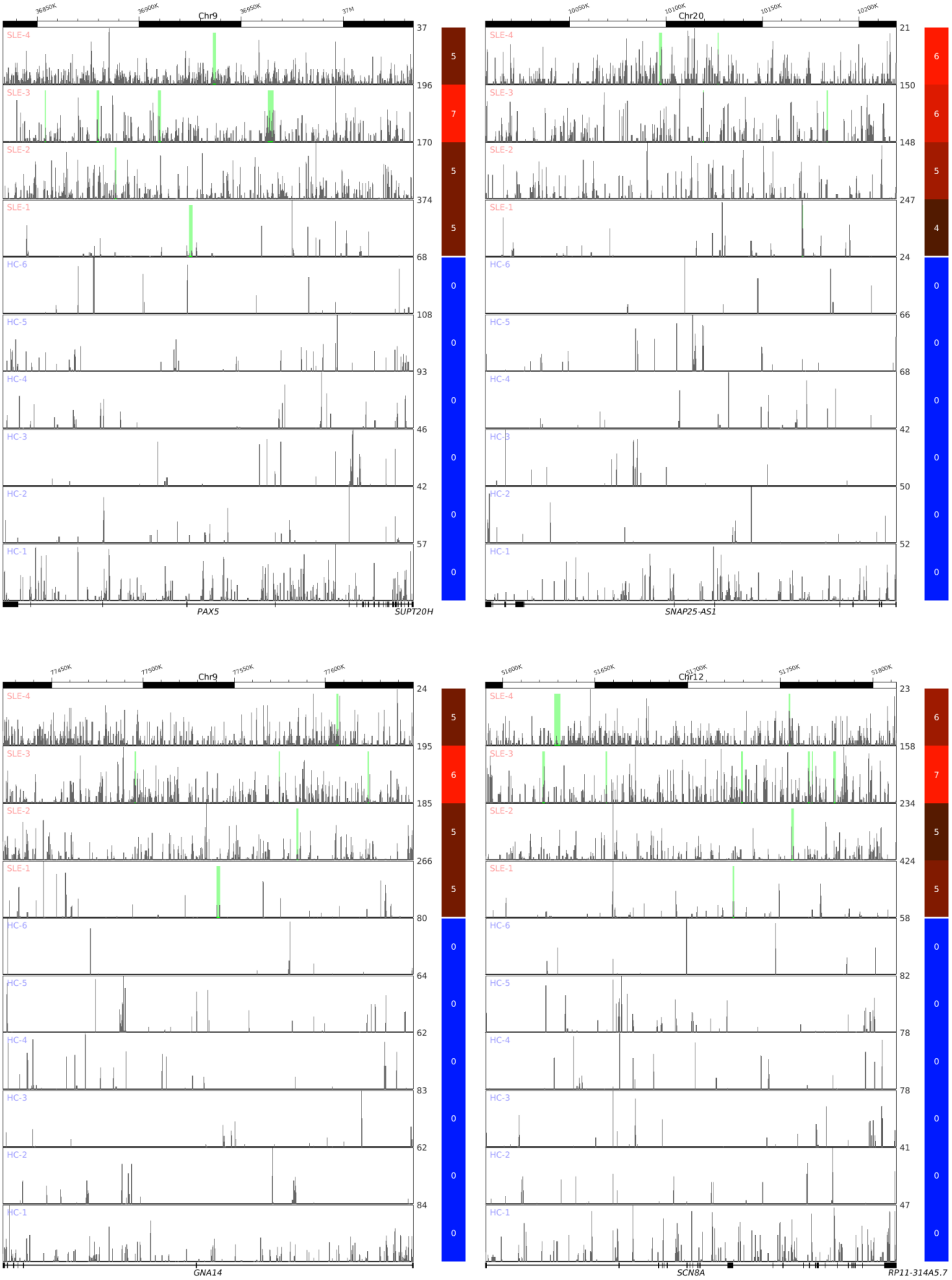
Chr9
36850K
36900K
36950K
37M
SLE-4
SLE-3
SLE-2
SLE-1
HC-6
HC-5
HC-4
HC-3
HC-2
HC-1
PAX5
SUPT20H
Chr20
10050K
10100K
10150K
10200K
SNAP25-AS1
Chr9
77450K
77500K
77550K
77600K
GNA14
Chr12
51600K
51650K
51700K
51750K
51800K
SCN8A
RP11-314A5.7

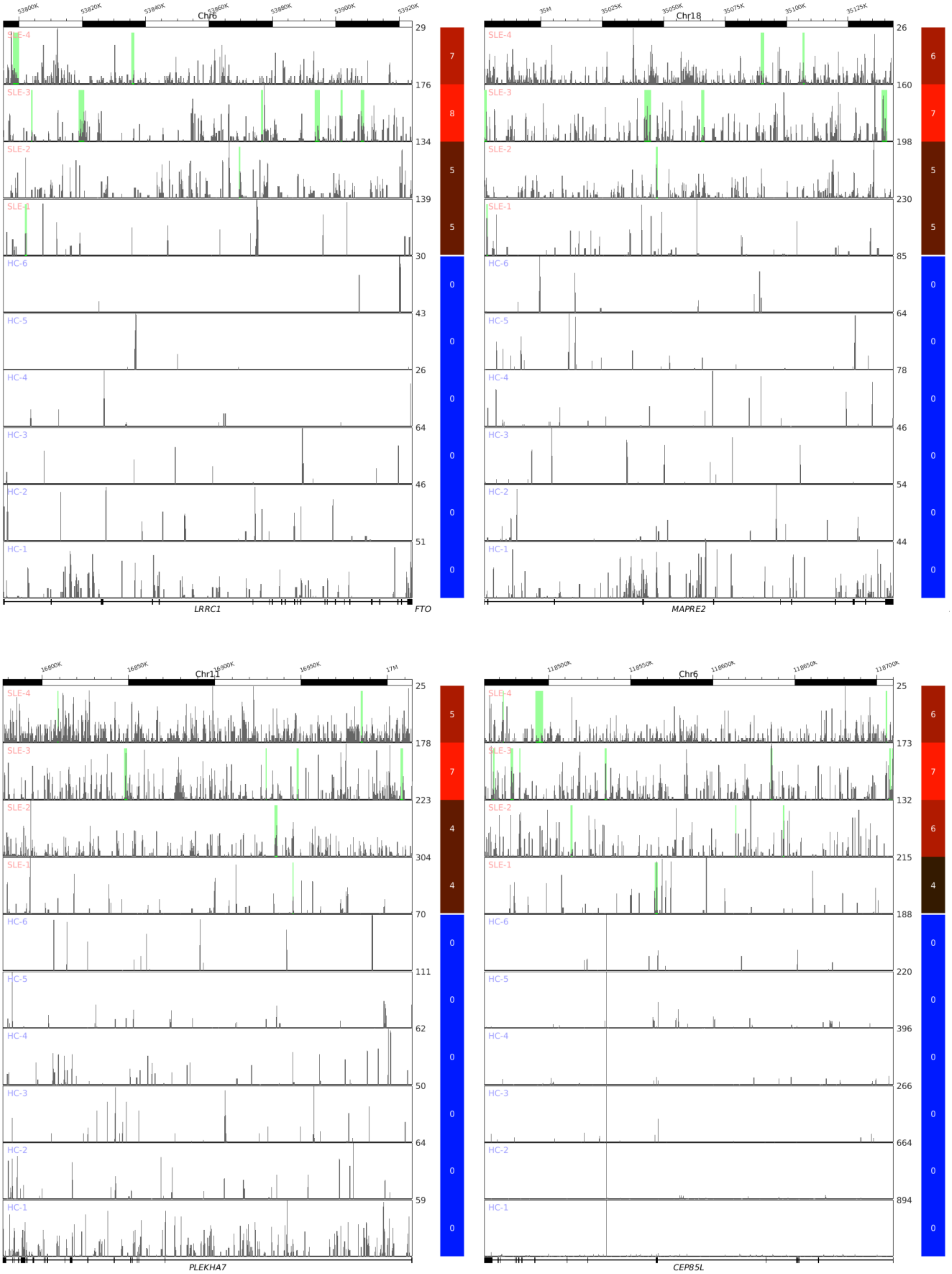

Chr6
53800K
53820K
53840K
53860K
53880K
53900K
53920K
SLE-4
SLE-3
SLE-2
SLE-1
HC-6
HC-5
HC-4
HC-3
HC-2
HC-1
29
176
134
139
30
43
26
64
46
51
7
8
5
5
0
0
0
0
0
0
LRRC1
FTO
Chr18
35M
35025K
35050K
35075K
35100K
35125K
26
160
198
230
85
64
78
46
54
44
6
7
5
5
0
0
0
0
0
0
MAPRE2
Chr11
16800K
16850K
16900K
16950K
17M
25
178
223
304
70
111
62
50
64
59
5
7
4
4
0
0
0
0
0
0
PLEKHA7
Chr6
118500K
118550K
118600K
118650K
118700K
25
173
132
215
188
220
396
266
664
894
6
7
6
4
0
0
0
0
0
0
CEP85L

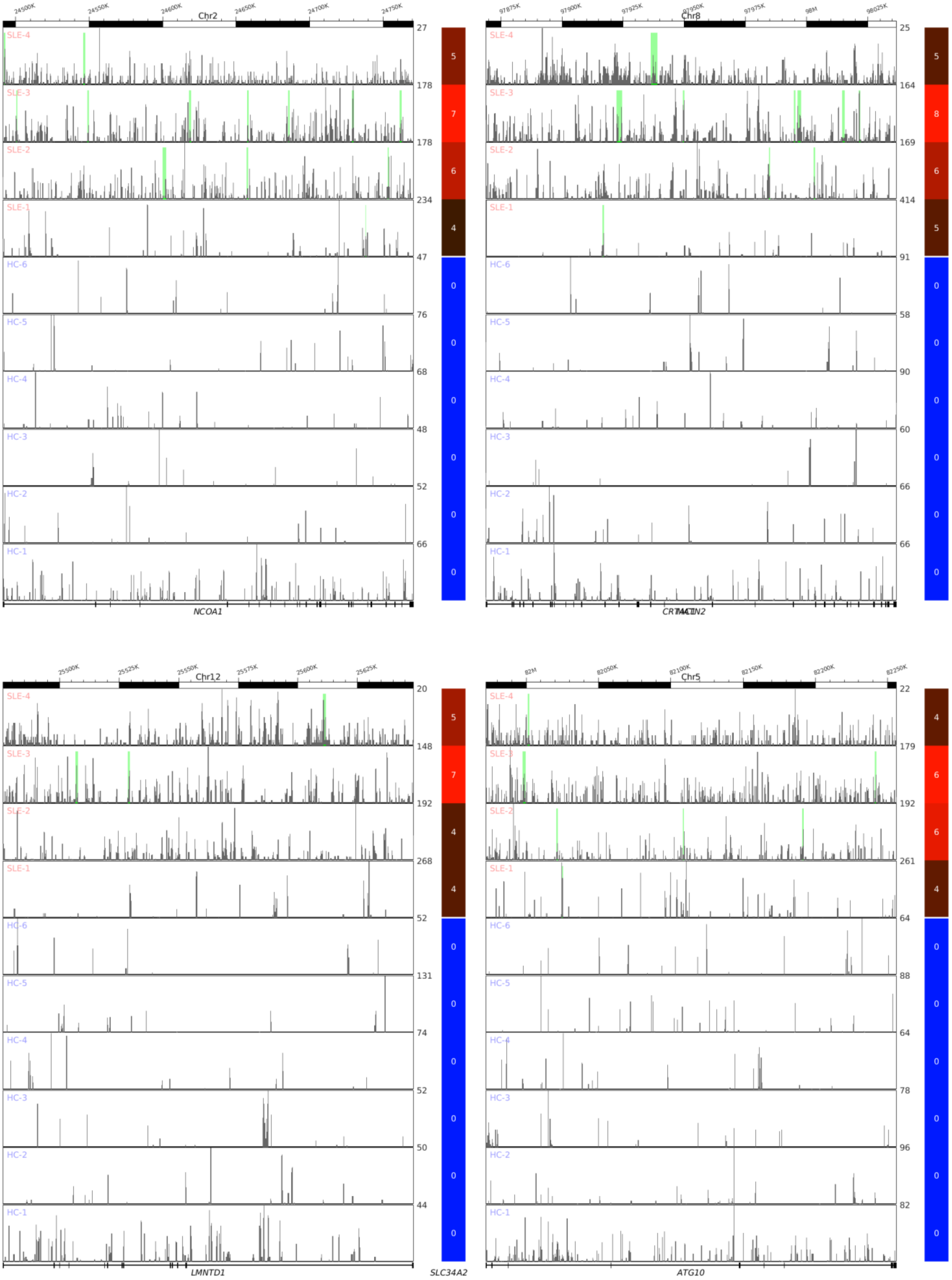

Chr2
24500K
24550K
24600K
24650K
24700K
24750K
SLE-4
SLE-3
SLE-2
SLE-1
HC-6
HC-5
HC-4
HC-3
HC-2
HC-1
NCOA1
Chr8
97875K
97900K
97925K
97950K
97975K
98M
98025K
Chr12
25500K
25525K
25550K
25575K
25600K
25625K
LMNTD1
SLC34A2
Chr5
82M
82050K
82100K
82150K
82200K
82250K
ATG10

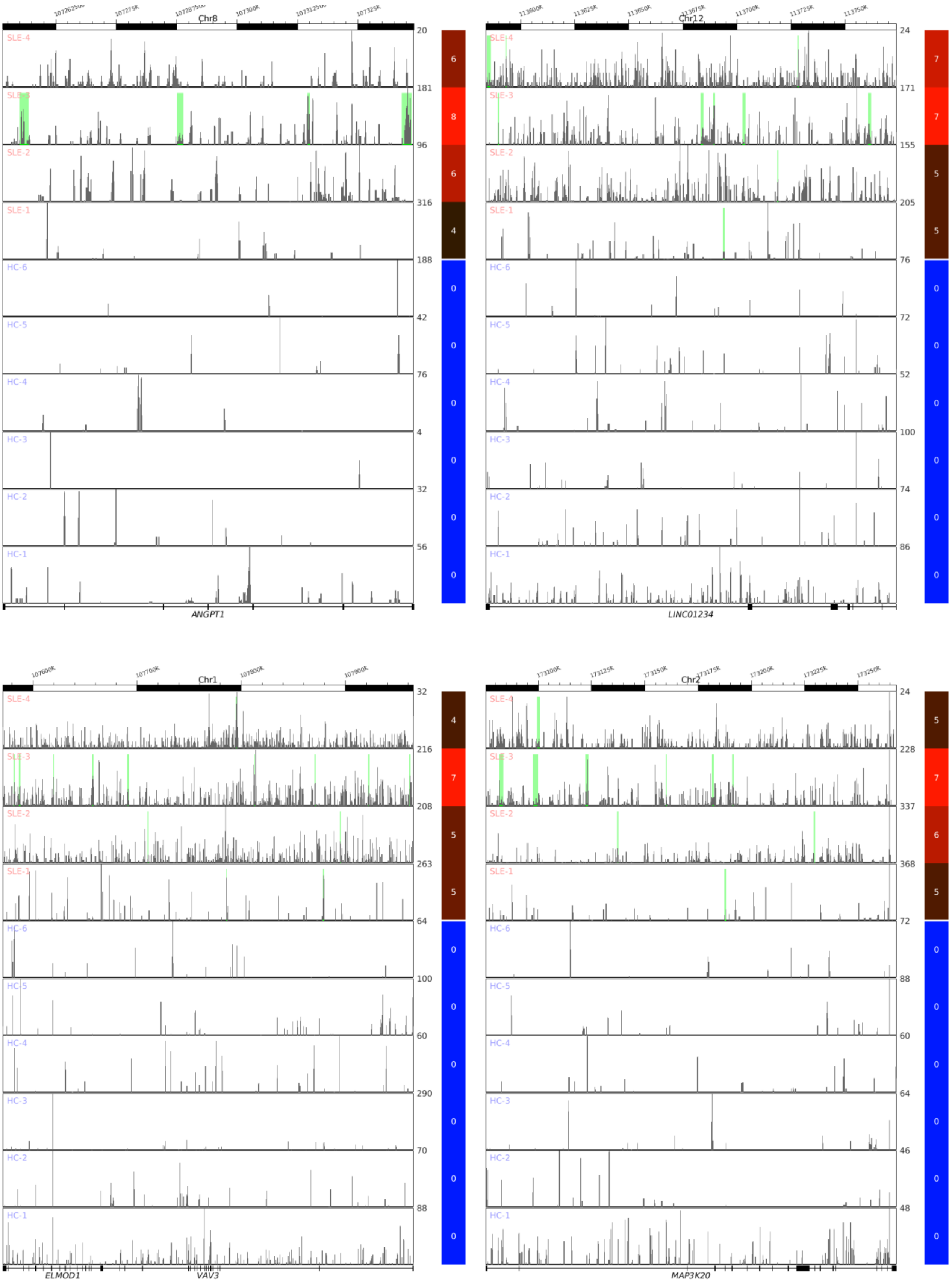
Chr8
SLE-4
SLE-3
SLE-2
SLE-1
HC-6
HC-5
HC-4
HC-3
HC-2
HC-1
20
181
96
316
188
42
76
4
32
56
6
8
6
4
0
ANGPT1
Chr12
113600K
113625K
113650K
113675K
113700K
113725K
113750K
24
171
155
205
76
72
52
100
74
86
7
7
5
5
LINC01234
Chr1
107600K
107700K
107800K
107900K
32
216
208
263
64
100
60
290
70
88
4
7
5
5
ELMOD1
VAV3
Chr2
173100K
173125K
173150K
173175K
173200K
173225K
173250K
24
228
337
368
72
88
60
64
46
48
5
7
6
5
MAP3K20

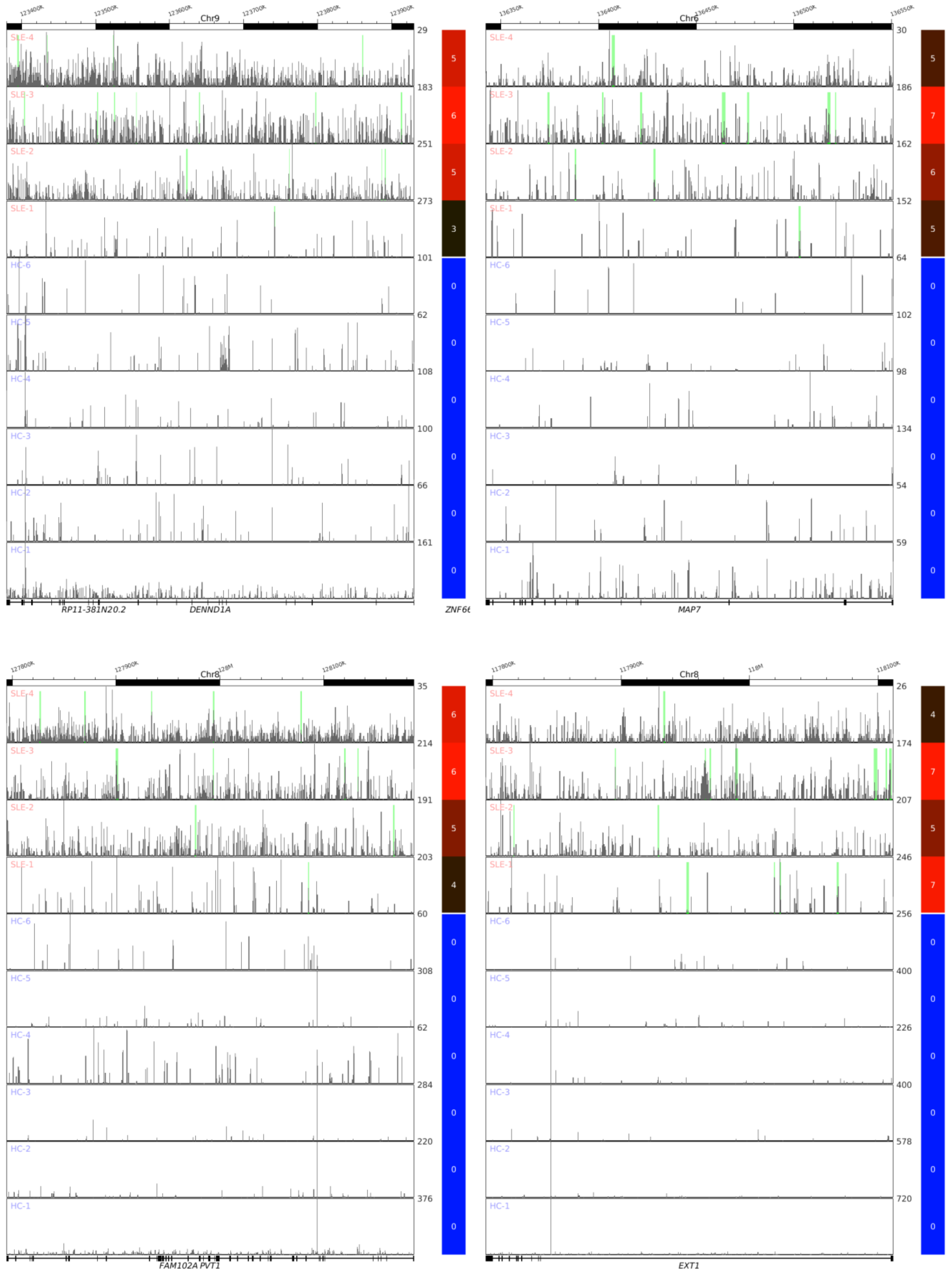

Chr9
SLE-4
SLE-3
SLE-2
SLE-1
HC-6
HC-5
HC-4
HC-3
HC-2
HC-1
RP11-381N20.2
DENND1A
Chr6
MAP7
Chr8
FAM102A PVT1
Chr8
EXT1

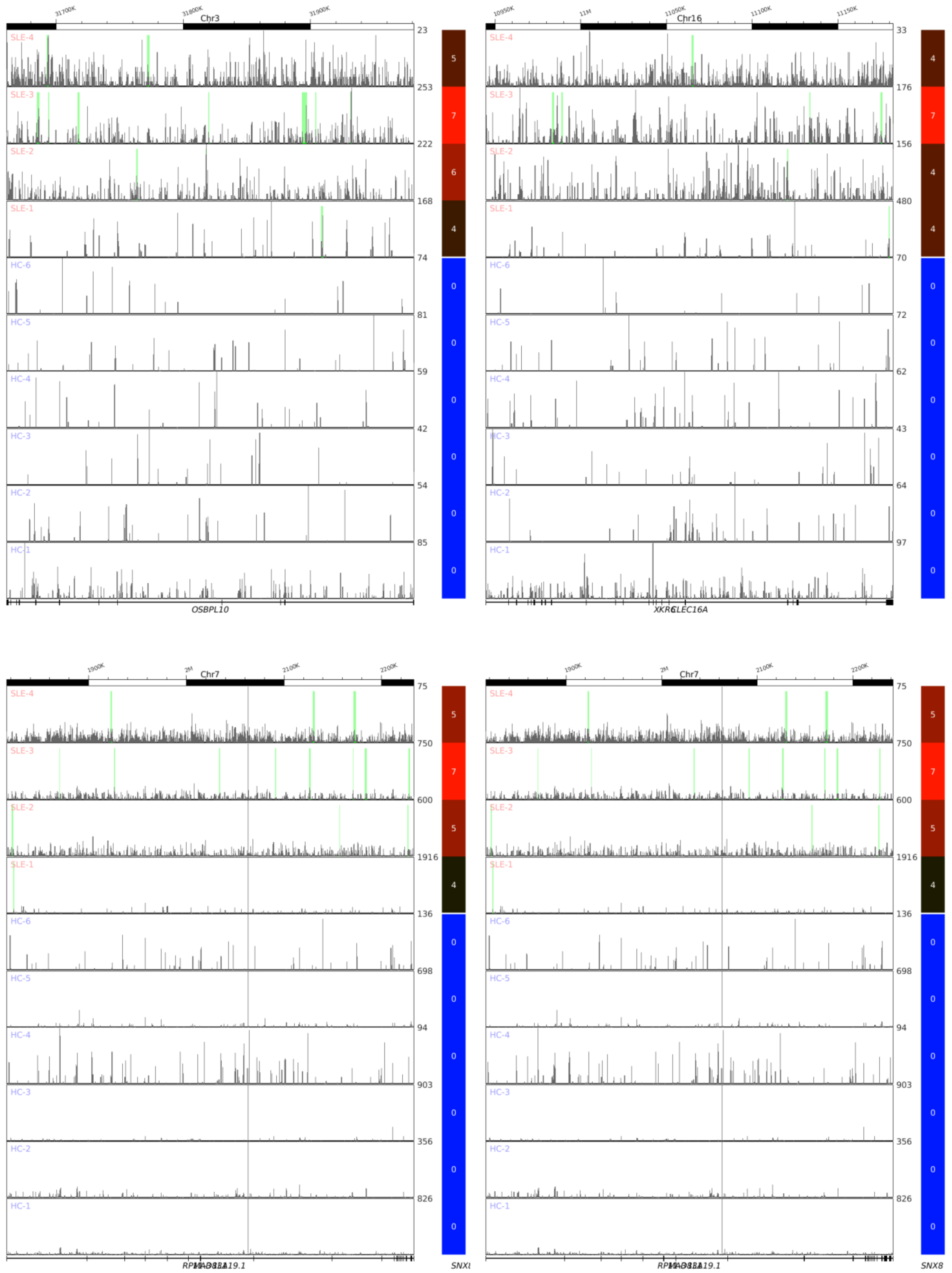

Chr3
31700K
31800K
31900K
SLE-4
SLE-3
SLE-2
SLE-1
HC-6
HC-5
HC-4
HC-3
HC-2
HC-1
OSBPL10
Chr16
10950K
11M
11050K
11100K
11150K
XKR6
CLEC16A
Chr7
1900K
2M
2100K
2200K
SNX8

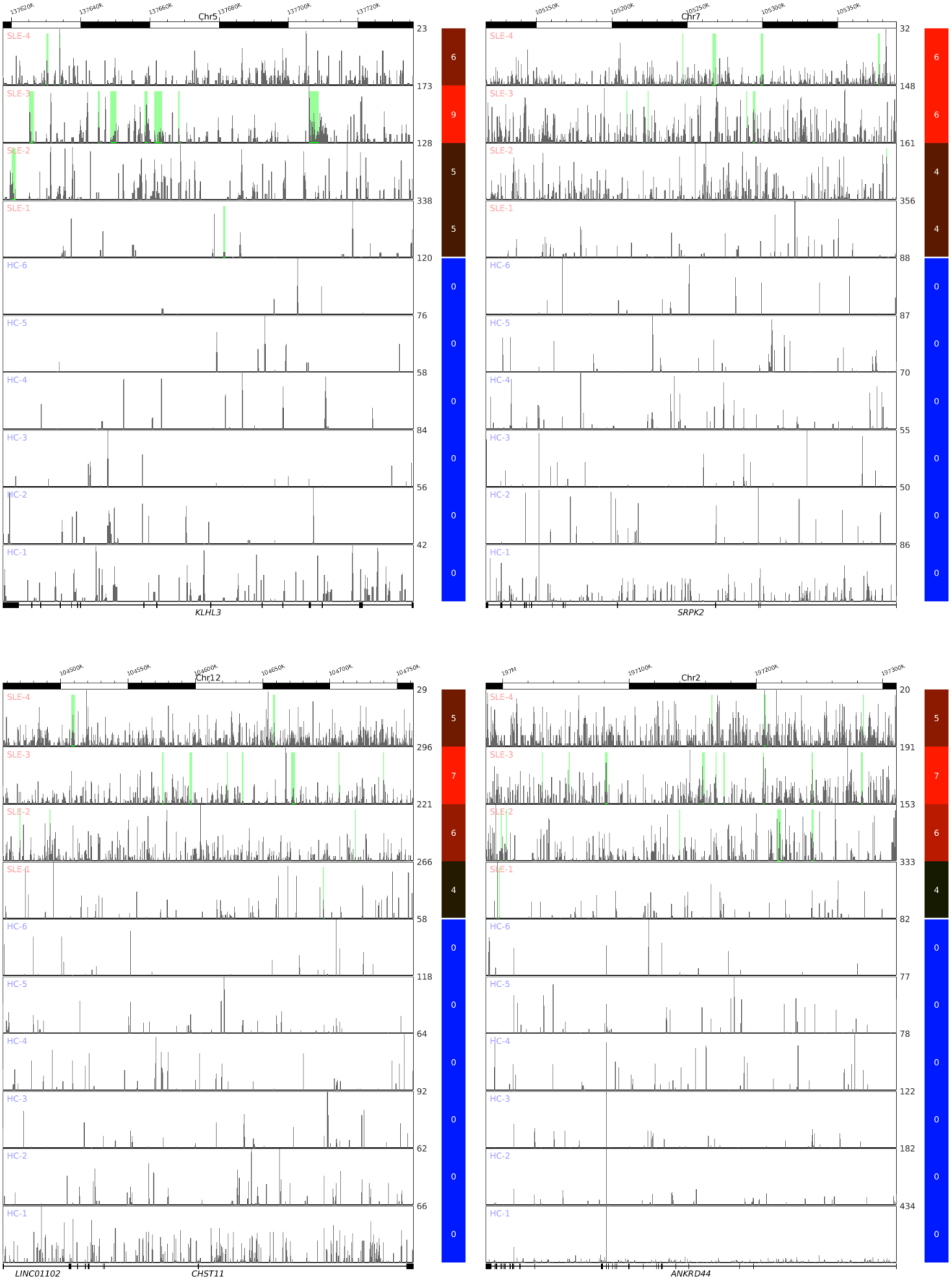
Chr5
137620K
137640K
137660K
137680K
137700K
137720K
SLE-4
SLE-3
SLE-2
SLE-1
HC-6
HC-5
HC-4
HC-3
HC-2
HC-1
23
173
128
338
120
76
58
84
56
42
6
9
5
5
0
0
0
0
0
0
KLHL3
Chr7
105150K
105200K
105250K
105300K
105350K
32
148
161
356
88
87
70
55
50
86
6
6
4
4
0
0
0
0
0
0
SRPK2
Chr12
104500K
104550K
104600K
104650K
104700K
104750K
29
296
221
266
58
118
64
92
62
66
5
7
6
4
0
0
0
0
0
0
LINC01102
CHST11
Chr2
197M
197100K
197200K
197300K
20
191
153
333
82
77
78
122
182
434
5
7
6
4
0
0
0
0
0
0
ANKRD44

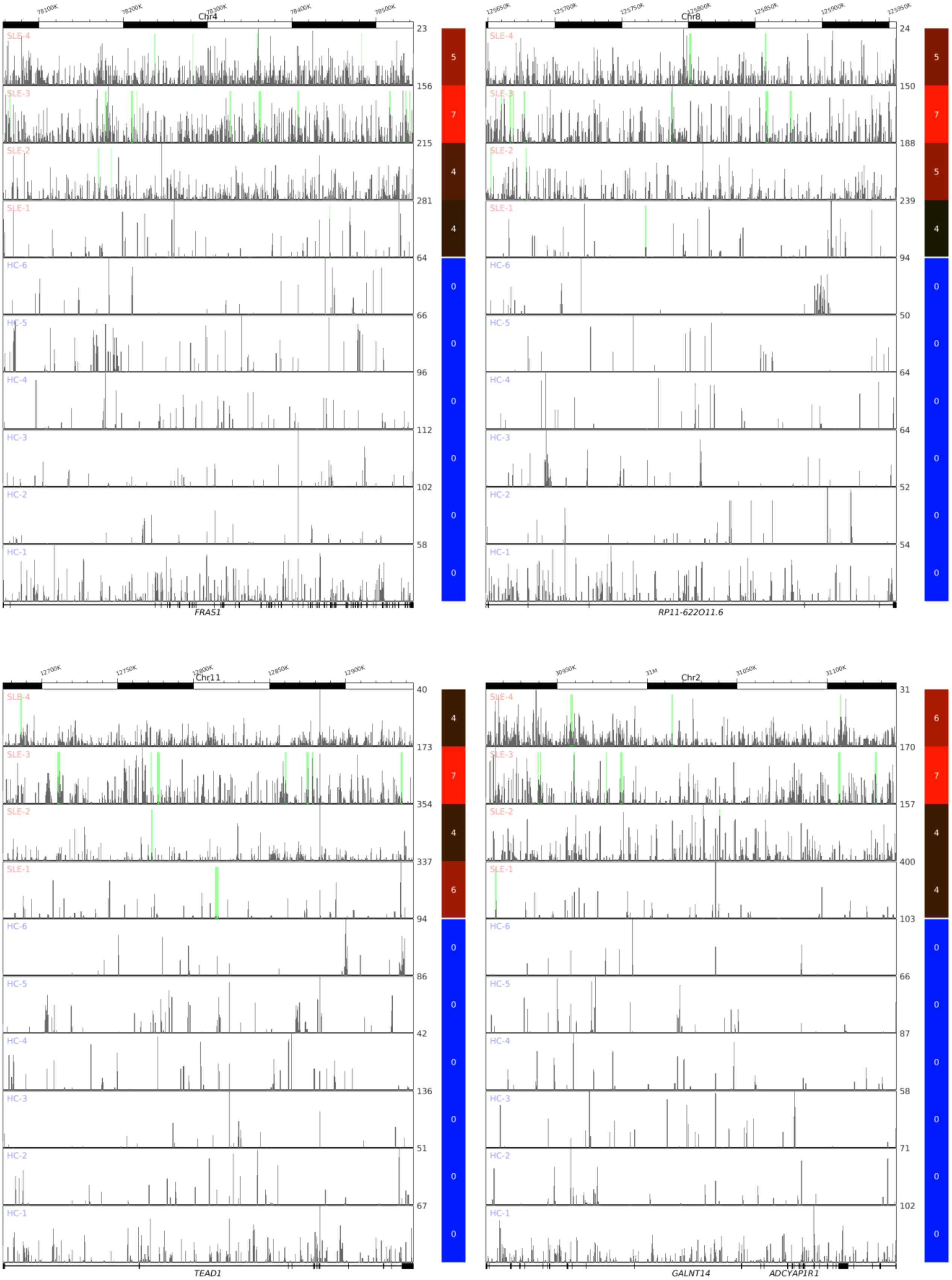

Chr4
78100K
78200K
78300K
78400K
78500K
FRAS1
Chr8
125650K
125700K
125750K
125800K
125850K
125900K
125950K
RP11-622O11.6
Chr11
12700K
12750K
12800K
12850K
12900K
TEAD1
Chr2
30950K
31M
31050K
31100K
GALNT14
ADCYAP1R1
SLE-4
SLE-3
SLE-2
SLE-1
HC-6
HC-5
HC-4
HC-3
HC-2
HC-1

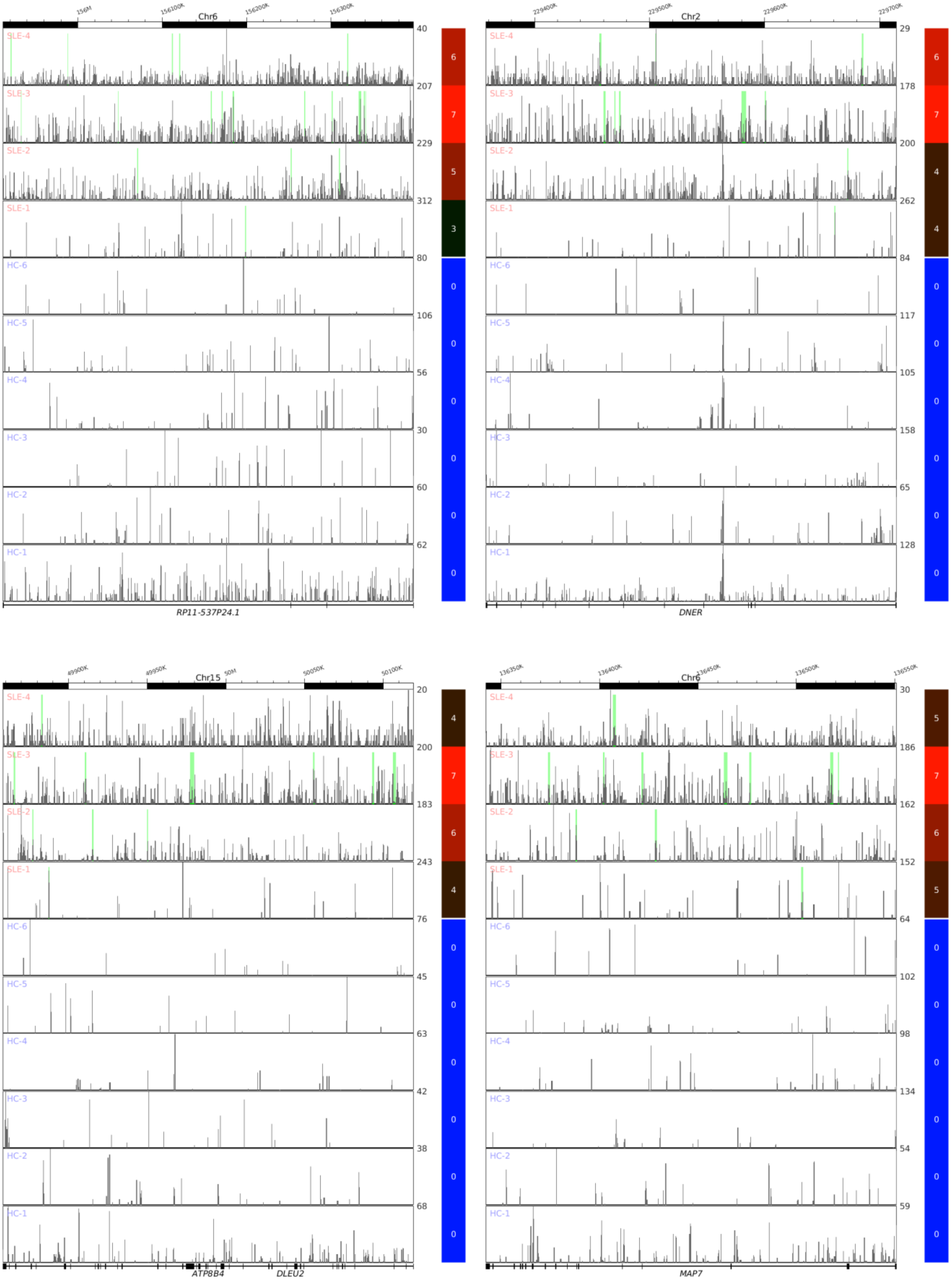
Chr6
156M
156100K
156200K
156300K
SLE-4
SLE-3
SLE-2
SLE-1
HC-6
HC-5
HC-4
HC-3
HC-2
HC-1
RP11-537P24.1
Chr2
229400K
229500K
229600K
229700K
DNER
Chr15
49900K
49950K
50M
50050K
50100K
ATP8B4
DLEU2
Chr6
136350K
136400K
136450K
136500K
136550K
MAP7

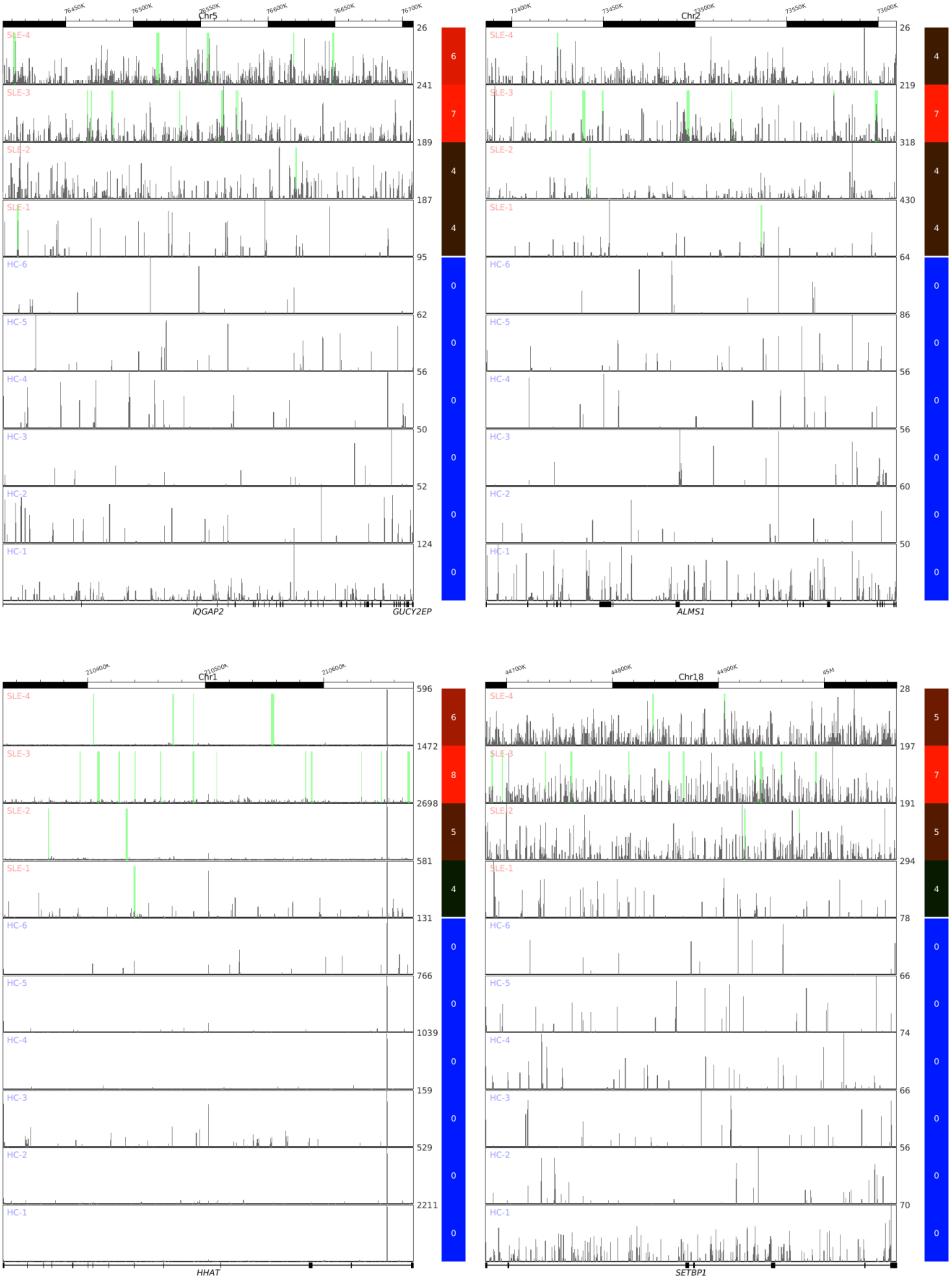

Chr5
76450K
76500K
76550K
76600K
76650K
76700K
IQGAP2
GUCY2EP
Chr2
73400K
73450K
73500K
73550K
73600K
ALMS1
Chr1
210400K
210500K
210600K
HHAT
Chr18
44700K
44800K
44900K
45M
SETBP1
SLE-4
SLE-3
SLE-2
SLE-1
HC-6
HC-5
HC-4
HC-3
HC-2
HC-1

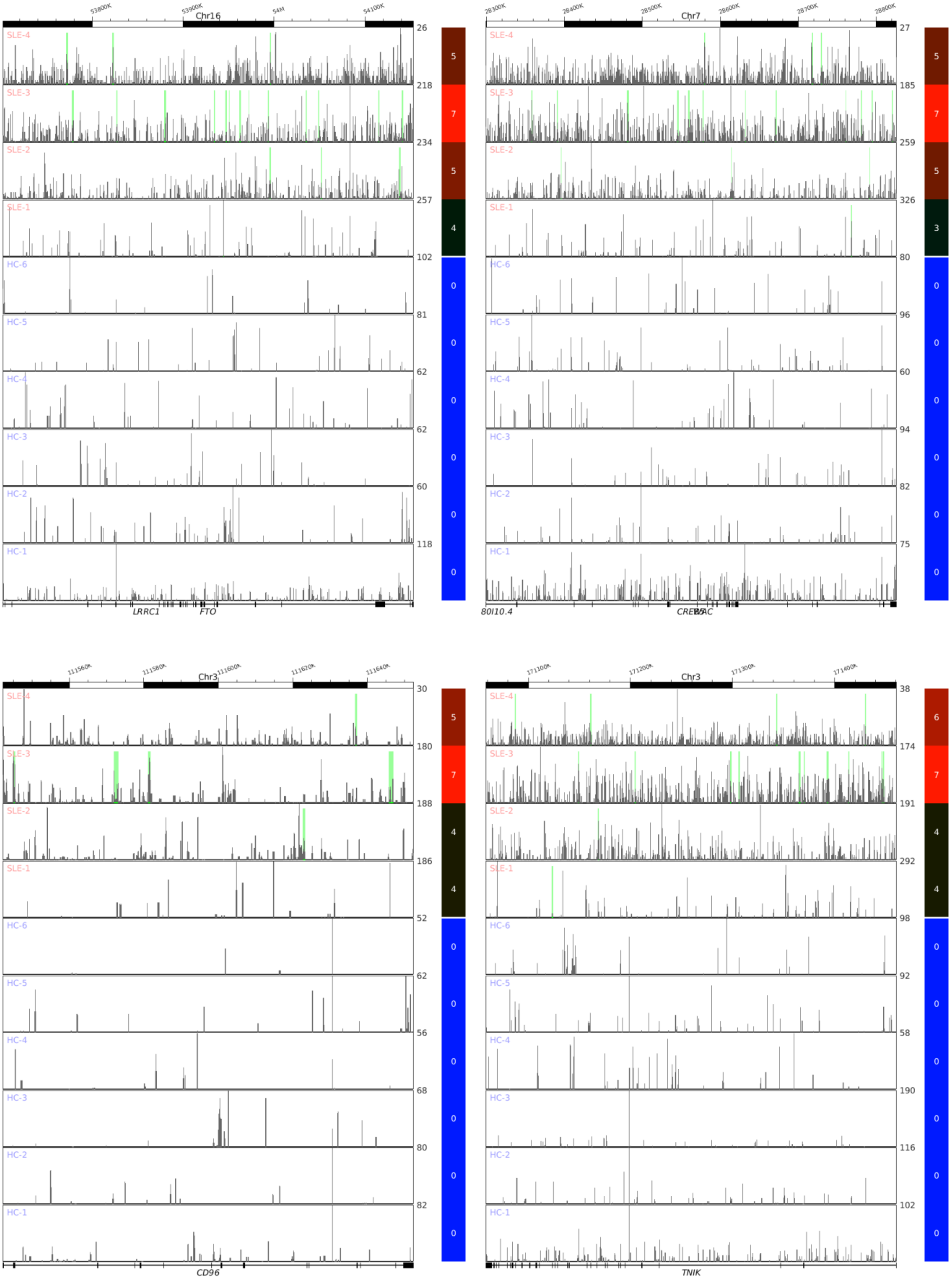

Chr16
53800K
53900K
54M
54100K
SLE-4
SLE-3
SLE-2
SLE-1
HC-6
HC-5
HC-4
HC-3
HC-2
HC-1
LRRC1
FTO
Chr7
28300K
28400K
28500K
28600K
28700K
28800K
80I10.4
CREB5
Chr3
111560K
111580K
111600K
111620K
111640K
CD96
Chr3
171100K
171200K
171300K
171400K
TNIK

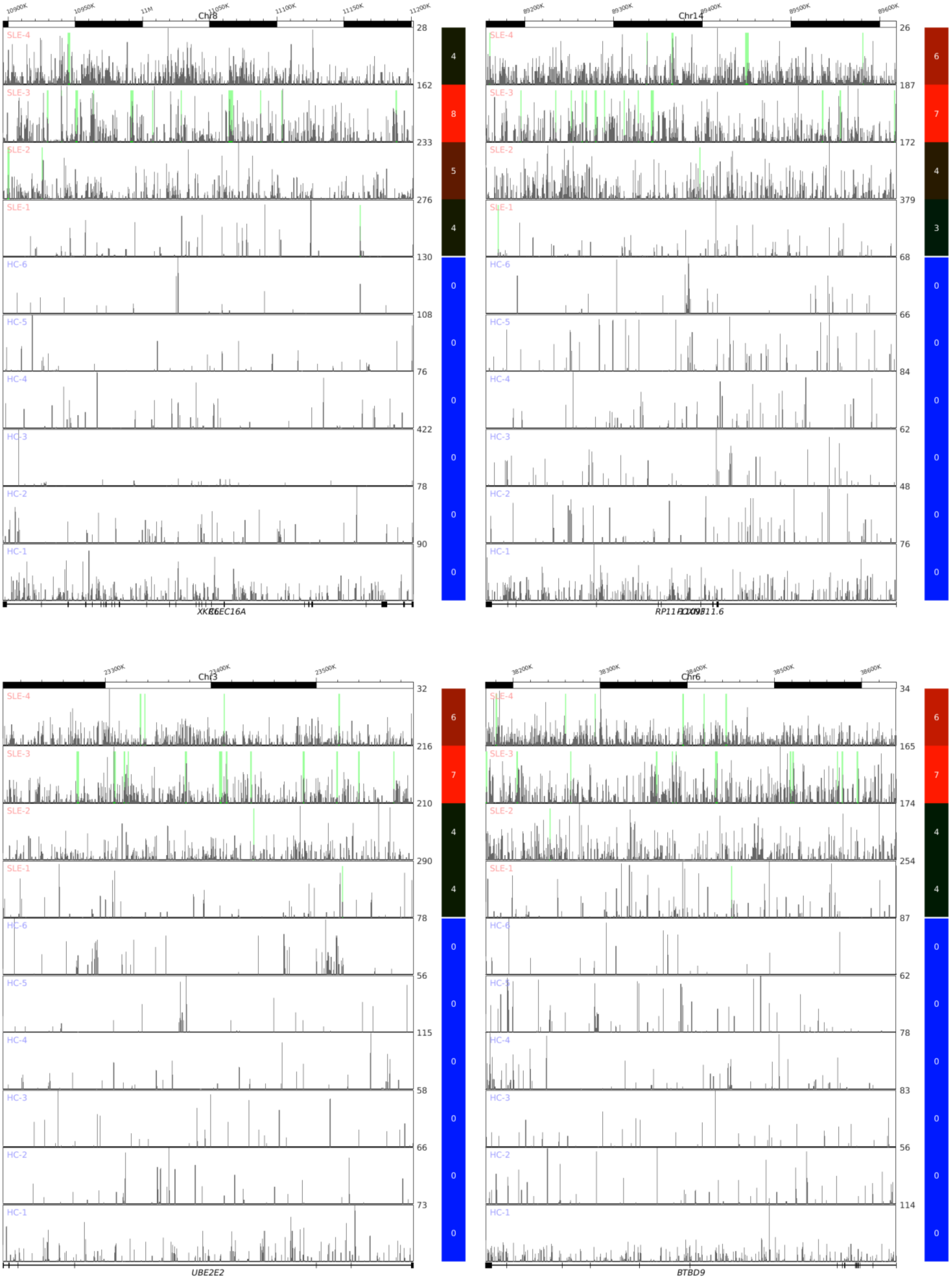
Chr8
Chr14
Chr3
Chr6
SLE-4
SLE-3
SLE-2
SLE-1
HC-6
HC-5
HC-4
HC-3
HC-2
HC-1
UBE2E2
BTBD9

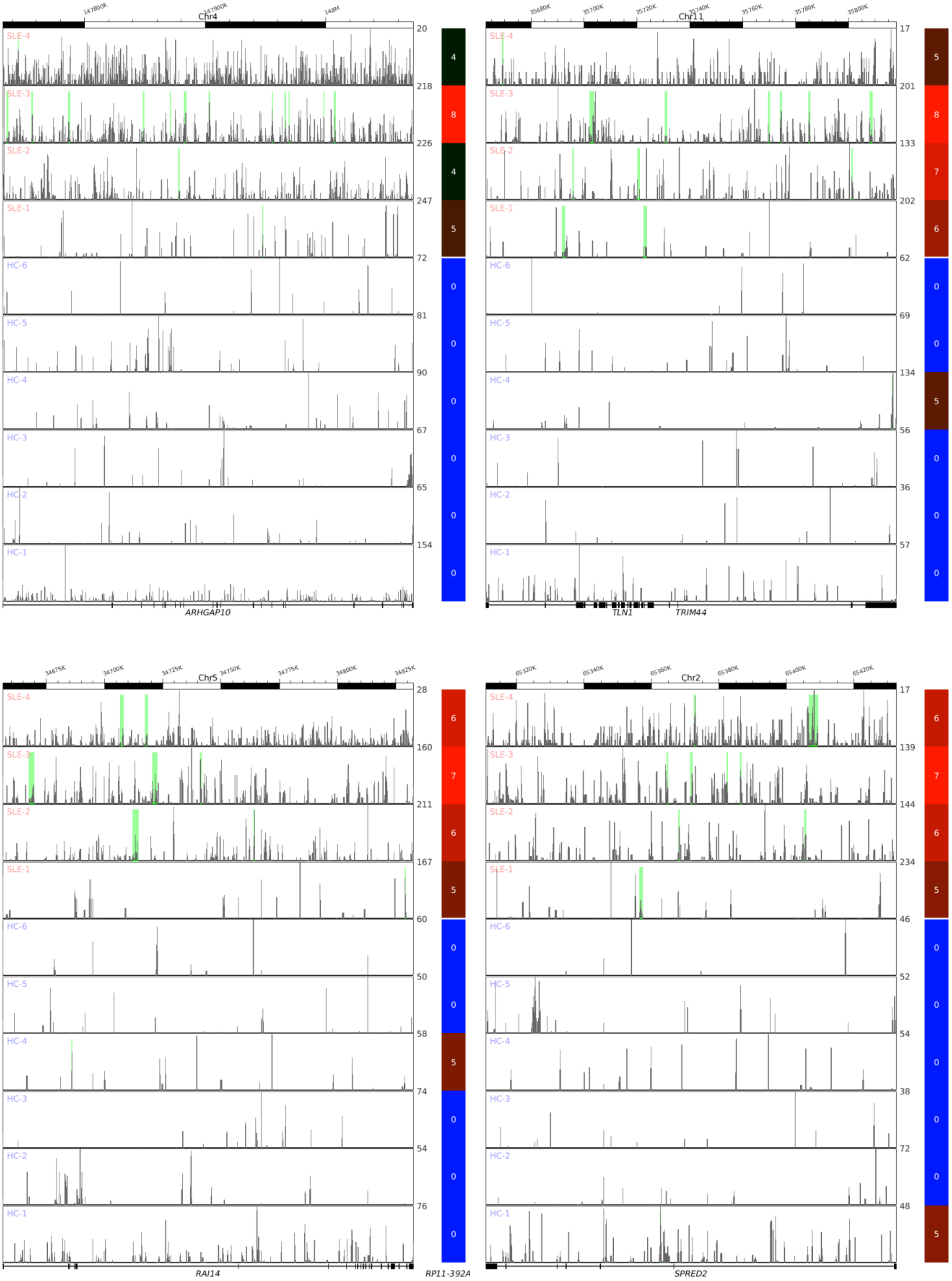

Figure S1. Track plots of the excision *loci* of the 96 top-ranked up-DPpGCs in systemic lupus erythematosus (SLE) with DNASE1L3 deficiency and healthy control (HC), and the corresponding gene coverage. Each horizontal line represents the length of a gene. The green bars represent the *loci* of the eccDNA. The color bars codify the scaled split read count of the eccDNA per gene in a $\log_2$ scale.
